# Local networks from different parts of the human cerebral cortex generate and share the same population dynamic

**DOI:** 10.1093/texcom/tgac040

**Published:** 2022-10-28

**Authors:** Alex Willumsen, Jens Midtgaard, Bo Jespersen, Christoffer K K Hansen, Salina N Lam, Sabine Hansen, Ron Kupers, Martin E Fabricius, Minna Litman, Lars Pinborg, José D Tascón-Vidarte, Anne Sabers, Per E Roland

**Affiliations:** Department of Neuroscience, Panum Institute, University of Copenhagen, Denmark; Department of Neuroscience, Panum Institute, University of Copenhagen, Denmark; Department of Neurosurgery, Rigshospitalet, University Hospital of Copenhagen, Denmark; Department of Neuroscience, Panum Institute, University of Copenhagen, Denmark; Department of Neuroscience, Panum Institute, University of Copenhagen, Denmark; Department of Neuroscience, Panum Institute, University of Copenhagen, Denmark; Department of Neuroscience, Panum Institute, University of Copenhagen, Denmark; Department of Neurosurgery, Rigshospitalet, University Hospital of Copenhagen, Denmark; Department of Clinical Neurophysiology, Rigshospitalet, University Hospital of Copenhagen, Denmark; Epilepsy Clinic, Department of Neurology, Rigshospitalet, University Hospital of Copenhagen, Denmark; Epilepsy Clinic, Department of Neurology, Rigshospitalet, University Hospital of Copenhagen, Denmark; Neurobiology Research Unit, Department of Neurology, Rigshospitalet, University Hospital of Copenhagen, Denmark; DIKU, Department of Computer Sciences, University of Copenhagen, Denmark; Epilepsy Clinic, Department of Neurology, Rigshospitalet, University Hospital of Copenhagen, Denmark; Department of Neuroscience, Panum Institute, University of Copenhagen, Denmark

**Keywords:** attractor properties, brain theory, cortical dynamics, fluctuations and oscillations, single trials

## Abstract

A major goal of neuroscience is to reveal mechanisms supporting collaborative actions of neurons in local and larger-scale networks. However, no clear overall principle of operation has emerged despite decades-long experimental efforts. Here, we used an unbiased method to extract and identify the dynamics of local postsynaptic network states contained in the cortical field potential. Field potentials were recorded by depth electrodes targeting a wide selection of cortical regions during spontaneous activities, and sensory, motor, and cognitive experimental tasks. Despite different architectures and different activities, all local cortical networks generated the same type of dynamic confined to one region only of state space. Surprisingly, within this region, state trajectories expanded and contracted continuously during all brain activities and generated a single expansion followed by a contraction in a single trial*.* This behavior deviates from known attractors and attractor networks. The state-space contractions of particular subsets of brain regions cross-correlated during perceptive, motor, and cognitive tasks. Our results imply that the cortex does not need to change its dynamic to shift between different activities, making task-switching inherent in the dynamic of collective cortical operations. Our results provide a mathematically described general explanation of local and larger scale cortical dynamic.

## Introduction

If collective operations of cortical neurons followed some principles, this would advance the understanding of the cerebral cortex. So far, no viable principles supporting collective behaviors of cortical neurons at a larger scale have been identified (Breakspear 2017; Buzsaki 2019; Singer et al. 2019; McCormick et al. 2020). One reason might be that operations in the cortex are high dimensional and complex. This complexity, however, does not exclude that diverse collective operations by larger populations of neurons are guided by simpler hidden dynamic principles. The purpose of this study was to determine if such hidden principles could be found in recordings from the human cerebral cortex.

Substantial parts of cortical operations are carried out by nonlinear postsynaptic operations of neurons in local cortical networks (Rheinberger and Jasper 1937; McCormick et al. 2020). The postsynaptic membrane currents of many neurons in a local cortical network collectively generate a field potential. The field potential consequently contains information of collective postsynaptic operations of a population of neurons at a larger, mm^3^ spatial scale (Einevoll et al. 2014; Pesaran et al. 2018). Local cortical networks thus contribute to cortical operations at this scale by changing the local states contained in their field potentials. Here, we examine whether postsynaptic states generated by the local networks follow certain general principles. To find such principles, one must reveal the state dynamics, i.e., reveal how the successions of states behave in state space. Here, we use the word state in its geometrical meaning: a point in state space.

There have been a vast number of studies of field potential oscillations. In these studies, two-dimensional field potential time series, under the assumption of stationarity, are separated and filtered into different frequency bandwidths of oscillations that are proposed to subserve different roles. However, this type of two-dimensional analysis or averaging data from single trials cannot capture the nonlinear dynamics of postsynaptic cortical states (Babloyantz and Destexhe 1986; Roschke and Basar 1990; Stam 1996; Rabinovich et al. 2012; Breakspear 2017; Davis et al. 2020). We therefore developed methods for deriving the local dynamics at their full multidimensionality directly from the measured field potential in single trials*.*

Different types of dynamical models have been proposed to explain the dynamics in field potentials during a few conditions in selected parts of the cortex. However, there has been no consensus of the dynamics generated by the different models (Tognioli and Scott Kelso 2011; Knierim and Zhang 2012; Rabinovich et al. 2012; Priesemann et al. 2013; Wimmer et al. 2014; Breakspear 2017; Hennequin et al. 2018). This could mean that different types of dynamics are reserved for different cortical functions. It could also mean that cortical networks, having different architectures, produce different dynamics. Here, we examine these 2 hypotheses.

As models do not reveal the dynamics hidden in field potentials, we took an experimental approach. Using depth electrodes, we recorded field potentials from many local cortical networks during a wide range of experimental conditions in patients with epilepsy and limited the analysis to recordings without any epileptic transients and epileptic activity.

The purpose of this study was neither to physiologically distinguish these behaviorally defined experimental conditions nor to map or functionally dissect each condition. Behavioral and psychological defined categories might not be the best descriptors for categorizing cortical operations (Buzsaki 2020). Despite this criticism, in most experimental studies, behavioral conditions are changed to induce changes in the measured variable, e.g., the field potential. Our study differs from such experimental studies*.*

Because the dynamics driving the field potential are hidden, they must first be extracted from the field potentials. Consequently, we first extracted and identified the dynamics of postsynaptic states contained in the field potentials with an unbiased method. Next, we examined whether different types of dynamics existed in the cortex at this spatial mm^3^ scale. Examples of different types of dynamics ruling complex systems are fixed points, bifurcations, low dimensional limit circles (oscillations), saddles, tori, attractor dynamics, strange attractors, meta-stability, and multistability (Rabinovich et al. 2012; Breakspear 2017). The many experimental conditions were included to reduce the risk of missing possible types of dynamics.

To our surprise, our results demonstrated that all local cortical networks produced the same type of dynamic confined to one region only of state space. In this region, the trajectories continuously expanded and contracted. Then, we examined whether this type of dynamics prevailed during all conditions, spontaneous activities, and tasks. It did. Finally, we examined whether this dynamic showed any modifications, which could relate to specific experimental activities. It turned out that subsets of local cortical networks adapted their state changes and mutually interacted by cross correlating their expansion and contraction of their trajectories in specific tasks. Our results deviate from earlier proposed dynamical models*.* In this sense, our results provide the first empirical evidence for a general dynamic governing local and larger scale cortical operations.

## Material and methods

A total of 13 patients (6 females, 7 males; age range: 19–55 years; mean: 38 years, SD: 12 years) participated in this study. The patients had drug resistant epilepsy, were candidates for surgical resection, and were implanted with depth electrodes, at locations in cerebral cortex determined to reveal the location of an epileptic focus and spread of epileptic discharges. The study was approved by the Human Ethics Committee for the Greater Copenhagen region (permission 56441), and consequently, written informed consent was obtained from all participants in this study. The patients spent from 5 to 13 days with the electrodes implanted.

**Fig. 1:**
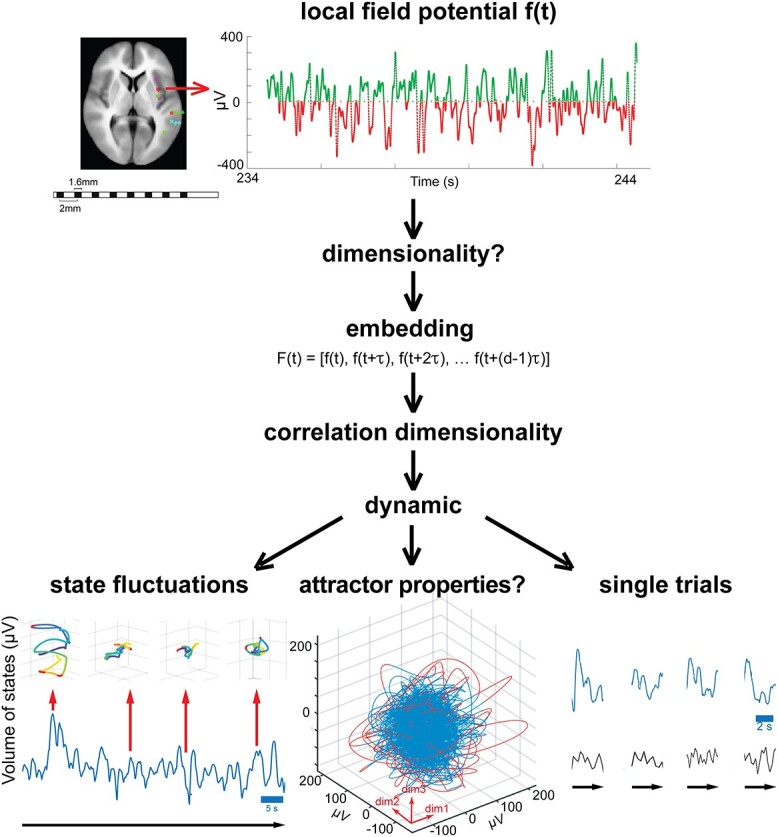
Logic of the analysis. Electrode lead with field potential *f*(*t*) sampled at 512 Hz. To find the dynamics of the local networks, we must know the *f*(*t*) dimensionality. We first constructed a vector *F*(*t*) of *f*(*t*) delayed by successive multiples of τ ms (embedding). The dimensionality of *F*(*t*) is the number of dimensions needed to achieve an exhaustive description of *F*(*t*). We then calculated the correlation dimensionality from this 20-dimensional vector *F*(*t*). We then studied the dynamic in state spaces of the found correlation dimension (ranging from 3 to 9). The dynamic consists of continuous state fluctuations, properties maintaining stability and single trial fluctuations. In state space, each point is one local cortical state. The points, 1.95 ms apart, are connected to form one blue—red colored trajectory (60 s long). When the trajectory escapes (red) from the blue dense part, it is rapidly pulled back. This resembles an attractor property, but the dynamics is not attractor dynamics because of the state fluctuations*:* These 50 ms volumes of evolving states fluctuate continuously, with consequent stretching and contraction of the state trajectories (the 4 examples show 1-s trajectories). Single trials: The volumes from a subset of leads (blue) show correlated expansion and contraction of their evolving state volumes, whereas other leads (black) do not. These properties roughly characterize the local cortical network dynamic.

The aim of this study was to obtain field potentials, without any abnormalities, from as many cortical sites as possible. The specific purpose of the study was to test if local cortical networks use different types of dynamics (fixed points, bifurcations, low dimensional limit circles (oscillations), saddles, tori, attractor dynamics, strange attractors, meta-stability, and multistability)*.* The experimental testing conditions were designed to be as diverse as possible in the clinical ward. The purpose of this diversity was to examine whether the dynamics driving the evolution of the field potential was different or explored different parts of state space. The experimental conditions were not designed to map or functionally dissect sensory, motor, and cognitive brain functions. The analysis design is summarized in Fig. 1.

After removal of all epileptic activity (see below), the material comprised 290 electrode leads. The anatomical locations of the electrode leads are listed in Table 1.

**Table 1:** Anatomical localization of the electrode leads.

Anatomical site	No leads
Hippocampus	38
head (20)	
body and tail (18)	
Middle temporal gyrus	35
anterior part (8)	
middle part (19)	
posterior part (8)	
Cingulate gyrus	29
anterior part (19)	
middle part (5)	
posterrior part (5)	
Insula	27
anterior part (13)	
posterior part (14)	
Superior temporal sulcus	18
anterior part (3)	
middle part (7)	
posterior part (8)	
Superior temporal gyrus	12
anterior + middle part (10)	
posterior part (2)	
Superior frontal gyrus	12
Parietal operculum	11
Temporal pole	10
Inferior temporal gyrus	10
Supramarginal gyrus	9
Fusiform gyrus	9
Superior frontal sulcus	9
anterior part (1)	
posterior part (8)	
Parahippocampal gyrus	8
Orbitofrontal cortex	7
lateral part (4)	
medial part (3)	
Middle frontal gyrus	5
Frontal operculum	5
Lingual gyrus	4
Entorhinal cortex	3
Inferior frontal gyrus	2
Precuneus	2
Gyrus precentralis	1
Gyrus postcentralis	1
Calcarine cortex	1
Angular gyrus	1
Subiculum	1

### Experimental conditions

Typically, 1–3 days after the implantation, the patient participated in different tests aimed to induce behavioral brain states as diverse as possible. In addition, we selected episodes of sleep, conversation with the nurse, TV watching, and eating. The purpose of the many behavioral conditions was to let the field potential dynamics explore state space over a wide range. For different practical reasons (medication, post-ictal sleep, early dismissal) not all patients performed all tests. Subjects were sitting or lying in bed. All behavioral conditions were collected several hours away from ictal and postictal activity, typically in the first 2 days in the ward.

#### Visual tests

We collected a series of 200 color pictures of landscapes and outdoor environments, devoid of images of humans (96 dpi, 1080 × 1920 pixels) downloaded from freeware sources on the internet. The pictures were presented on a screen measuring 53 × 30 cm (refresh rate: 144 Hz) which was placed ~120 cm from the eyes. Patients were instructed just to watch; with the exception of visual test 6, no behavioral responses were required. A total of 11 visual tests was conducted.

*Visual test 1*: 200 pictures appeared, 1 frame each (6.94 ms) with no intermission.

*Visual test 2*: 100 pictures were randomly selected and each was presented for 49 ms with no intermission.

*Visual test 3*: the same 100 pictures were presented each for 125 ms with no intermission.

*Visual test 4*: the same 100 pictures were presented each for 125 ms, followed by a 999-ms lasting black screen.

*Visual test* 5: the same 100 pictures were presented each for 999 ms, followed by a 999-ms lasting black screen.

*Visual test* 6: The next day, the 100 pictures were mixed with 100 new ones that the patient had not seen before. The 200 pictures were randomly presented each for 999 ms, followed by a 1999 ms lasting black screen. Following each picture, the patient had to say yes if he/she had seen it earlier (recognition test).

*Visual test* 7: apparent motion. A 3° × 3° black square moved on a white background in steps of 2° from the left to the right border of the screen, in 583 ms. The test was repeated 100 times.

*Visual test* 8: same as visual test 7, but here the square moved from right to left.

*Visual test* 9: one stationary 3° × 3° white square was shown for 1 min.

*Visual test* 10: one 3° × 3° red square, flanked by four 3° × 3° green squares, was shown for 2 min.

*Visual test* 11: one white square, 3 white squares in a row, one red square flanked by 4 green squares, or one green square flanked by 4 red squares were shown for 250 ms. Presentation was random and balanced such that each alternative was shown 50 times. All squares measured 3° × 3°.

#### Ganzfeld and rest condition

In the Ganzfeld condition, the patients’ eyes were occluded for 10 min by white ping pong balls, cut in halves, and covered by surgical gauze pads secured by tape, preventing any patterned visual input. The patients were asked to keep eyes open during the test. In the rest condition, the patient had eyes closed, was encouraged to relax, concentrating on having it black in front of the mind’s eye, but stay awake. Patients were supine in both control conditions.

#### Emotions

A total of 35 pictures (72 dpi) were taken from the Karolinska Directed Emotional Faces test (Lundqvist et al. 1998), showing 7 different emotional expressions (angry, disgusted, sad, fearful, happy, surprised, and neutral). Each image was shown for 6 s, followed by a 2-s dark screen. Following each picture, patients were asked to tell the type of emotional expression.

#### Memory retrieval tests

During these tests, the patient had eyes closed.

*Memory test 1*: recall faces. With 5 s nterval, the experimenter read the names of 29 well-known persons or cartoon characters (e.g. queen Margrethe, Donald Duck, Angela Merkel). The patient responded by yes or no whether he/she could clearly visualize the face.

*Memory test 2:* recall places. With 5 s interval, the experimenter read the names of 15 well-known landmarks or tourist attractions (e.g. the little mermaid, golden gate bridge, the Chinese wall). The patient responded by yes or no whether he/she could clearly visualize the place.

*Memory test 3:* mental navigation. The patient was asked to imagine going out of the front door of his/her residence, going to the right down the street, then walking down the first street appearing to the left and then alternatively to the right and left as in (Roland and Friberg 1985). After 65 s, the patient was asked to tell where she/he is.

*Memory test 4:* same as 3, but starting taking the home street to the left (Roland and Friberg 1985).

*Memory test 5:* a known target at some distance from the patient’s house was chosen. The patient was asked to imagine walking/cycling slowly to the target and say “now” upon arrival.

*Memory tests 6 and 7:* same as memory test 5 but each with a new target.

*Memory test 8:* patient was asked to imagine standing at target (condition 5) and walking/cycling back home. Upon arrival, patient says “now.”

*Memory test 9:* The patient imagines standing at target in condition 6, otherwise as in condition 8.

*Memory test 10*: patient was asked to recall all events in temporal order during the hospital admission day, from getting out of bed in the morning until arriving at the hospital. Upon arrival, patient says “now.”

#### Classification of nouns

A list of 19 Danish nouns all starting with “hoved” (head or main in English) was read (6 s interstimulus interval), and the patient was asked to classify each noun as being “concrete” or “abstract.”

#### Motor tests

An EMG disc electrode was fixed to the skin over the forearm extensors, contralateral to the brain hemisphere carrying the largest number of implanted electrodes. A reference electrode was fixed near the wrist, and the EMG signal was sampled by an EMG amplifier (10 Hz–5 kHz band-pass filter; amplified and finally recorded at 30 kHz) and used to define the motor epochs.

*Motor test 1*: the patient was asked to move the arm with the electrode such that the hand touches the left chest, to move it back to the original rest position, and then to touch the right side of the chest. Then, in similar order, the same hand moves to touch right and left shoulder, right and left foot, right and left knee, right and left abdomen, forehead, right and left heel, right and left thigh, opposite elbow, and opposite hand, in total lasting 60 s.

*Motor test 2*: the patient was asked to quickly bend the index-finger and thumb to touch, then quickly to extend the fingers again and to continue this as quickly as possible for 60 s.

*Motor test 3*: as in motor test 2, but synchronously with the index and thumbs of both hands.

*Motor test 4*: as in motor test 3, but counterstroke.

*Motor test 5*: rhythm. With the hand contralateral to the hemisphere with the majority of implanted electrodes, the index touches the thumb for approximately one s, followed by 3 short touches and so on in the same rhythm for total of 60 s.

*Motor test 6*: With the same hand as condition 5, the thumb touches shortly the index, middle finger, ring finger and little finger, the little finger, ring finger, middle finger and index, and so back and forth for total of 60 s.

#### Attention tests

*Somatosensory attention*: the patient expected a touch stimulus close to detection threshold with a thin calibrated filament (pressure of 10–20 mg) on the tip of the index finger. The stimulus was first presented after 60 s had elapsed (Roland 1981).

*Auditory attention*: The patient was asked to listen to a list of random numbers between 1 and 100, pronounced close to his/her auditory threshold. The patient had to repeat each detected number. In the ensuing 60 s period, no numbers were spoken. After 60 s have elapsed, a faint but detectable number was spoken, as a control.

#### Stereognosis

A small object (e.g. a cap of a USB-stick) was placed in the palm of the patient’s hand. The patient had eyes closed and was prompted to manipulate the object with the fingertips. The testing lasted up to 30 s, or until the object was recognized. The test was then repeated with the other hand with another object.

#### Smell and taste

Ortho-nasal smell was tested according to conventional clinical bedside tests using mild odorants such as tea and chocolate. The patient was instructed to sniff deeply, with eyes closed, while the odorant was held close to patient’s nose. Five odorants were used consecutively. Each odorant was tested for 20 s or until the odor was identified, whichever came first.

Retro-nasal smell and taste were tested by the patient pinching the nose with one hand, while he/she placed a sweet in the mouth with the other hand. Mouth-breathing was allowed. The patient was instructed to swallow only saliva while chewing gently on the sweet. The patient was asked to report the taste of the sweet. After 20 s, the patient was instructed to exhale through the nose and report any changes in the “taste” (flavor) of the sweet.

All patients understood the task instructions and performed the tests correctly*.*

#### Sleep

1. Light sleep. Visual confirmation of sleep spindles, 9–16 Hz, minimum duration 0.5 s, maximum 2 s, appearing more frequently than 0.3 Hz in the field potential time series. In addition, visual confirmation of K-complexes >200 μV, duration >200 ms, diphasic configuration. Finally, the selected 60 s episode should have maximum 20% delta activity. Light sleep was defined as a period of 60 s with at least 2 sleep spindles and 1 K-complex. Sleep spindles and K-complexes were detected by an algorithm and afterwards visually confirmed (Andrillon et al. 2011). The criterium for sleep spindles was an increase in 9–16 Hz activity of at least 3 SD, with a minimum duration 0.5 s, and a maximum 2 s.
2. Slow wave sleep. The episode selected had more than 60% slow waves, 1–2 Hz > 150 μV peak-to-peak. The amplitudes of the slow waves were more than 3 SD of 0.1–2 Hz activity and had peak-to-valley of at least 150 μV occupying more than 20% of the 60 s period (Hori et al. 2001). All episodes selected were visually confirmed as slow wave sleep. (Sleep was not the particular focus of this study, examples of light and slow-wave sleep are in **additional supporting files**  https://drive.google.com/drive/folders/1Fi44M3Xt_HwkId21EWF3hSOh6Peb0WYt?usp=sharing)

### Field potentials

The recordings were made with shielded cables and connection to a common ground. Field potentials were sampled at 512 Hz with a band-pass filter of 0.1–256 Hz from the implanted electrodes (SEEG electrodes; Ad-tech Medical Instruments, 1.28 mm in diameter, lead length 1.57 mm) in 13 patients. The field potential electrode leads had low impedance, a few Ohms (Misra et al. 2014)*.*

The electrode locations, based exclusively on clinical criteria, were determined from the post-operative CT-scan (1 mm^3^ voxels) co-registered to the patient’s *T*_1_-weighted magnetic resonance image (MRI) of the brain.

The sources of the field potential are the transmembrane currents *I_n_*(*t*).

From (Lindén et al. 2010):

(1) \begin{equation*} f(\boldsymbol{r},t )=\frac{1}{4\pi \sigma}\sum_{n=1}^N\frac{I_n(t)}{\left|\boldsymbol{r}-{\boldsymbol{r}}_n\right|} \end{equation*}

*f*(*t*) is the field potential at a particular instant for a population of neuron membranes close to the electrode lead (Pettersen and Einevoll 2008; Lindén et al. 2010). *I_n_*(*t*) is the net transmembrane current at a particular distance ***r****_n_* from the lead. Under the assumption that the extracellular conductivity, σ, is constant over the whole range of the spatial summation in equation (1), *f*(*t*) at each electrode lead, is the weighted (by their inverse distance from the lead) sum of the membrane and extracellular currents in the sampling space.

Electrodes with leads picking up noise were eliminated from the recordings (reasons: usually because of a gap in connections). Artifacts (synchronous in all leads) and episodes with noise synchronous over several leads were removed. Low-frequency drifts were removed by the hardware filters on the AD-converter. This was usually sufficient to remove any form of technical noise. In addition, the common reference to all remaining leads efficiently reduced the influence of remote sources on the local field potential*.*

The field potential may show net increase of inward currents similar to a net decrease at another electrode, because polarity depends on the reference and might be reversed in recordings. Similarly, inward currents and outward currents in the space sampled by the electrode might cancel, despite lively synaptic activity (Einevoll et al. 2014; Pesaran et al. 2018). After removing the artifacts and noise, we used a common reference for each of the gray matter leads. The common reference was the median of the signal in all intracranial leads. This efficiently reduces the influence of remote sources on the local field potential. Each field potential then is a time series of fluctuations around an overall median (Fig. 1). After this, the continuous data were reduced to 60 s epochs of field potentials representing the first 60 s of every single experimental condition and electrode lead, in total 12949 60 s files. We then removed all leads outside gray matter.

#### Detection and removing interictal epileptic activity from data

From the continuous recordings of the field potential, all data earlier than 24 h after the electrode insertion were excluded. Data during and 12 h after a generalized tonic–clonic seizure, during and 8 h after a focal clinical seizure, and during and 2 h after purely electrographic seizures were excluded (Fraucher et al. 2018). All interictal epileptic spikes and sharp waves were identified by their criteria (de Curtis and Avanzini 2001)*.*

To identify interictal epileptic transients in the raw field potential, we used the Mexican hat Wavelet method of Hein and Tetzlaff (2005). We performed a wavelet transform on the raw field potential and applied a threshold to the transform to identify epileptic transients. Transient epileptic events were identified using a Mexican hat wavelet (from Matlab version 2018b). We used 3 wavelets with half widths of 0.02, 0.04, and 0.1 s). These wavelets were then convolved with the field potential signal to generate a time series where the amplitude correlates with transients in data with temporal characteristics of interictal activity.

We squared the convolved signal in these 60 s files and defined a maximum threshold of *X* 10^6^ μV^2^ as the limit of normal activity. Different thresholds of *X*, 0.8, 0.7, 0.5, were applied until a threshold was found to locate all epileptic spikes and sharp waves in the 60 s epoch files of 2 patients (4 and 17)*.* From these 2 patients, we examined the files without detected epileptic spikes and sharp waves*.* The visualization software automatically identified peak to peak amplitudes exceeding 95 and 99% in each file (but still less than the maximum threshold). These potentials were visually scrutinized for being spikes or sharp waves. The *X* threshold associated with lack of interictal activity in these 2 patients was then applied to 400 similar 60 s files from the remaining 11 other patients. The final chosen threshold of 0.5 10^6^ μV^2^ removed all epileptic spikes and sharp waves in tested files. Each located spike or sharp wave detected with the final threshold was replaced by a horizontal 1-s segment. Then, all files with one or more horizontal 1-s segments, i.e. with detected interictal epileptic activity, were removed from the original set of 12949 f(t) files at 476 cortical locations. This left 3476 field potential files from 290 cortical locations, each 60 s long. These 3476 files are in the supporting data repository*.*

### Reconstruction of the dynamics (embedding)

We assume that the dynamics of a local network in the cerebral cortex can be captured from a field potential recording. The dynamics are the set of rules by which the field potential evolves. For a given behavioral condition, the sampled field potential is a time series that one can examine in state space to reveal its dynamics. We reconstructed the behavior in state space from the field potentials from the 290 cortical electrode leads for each of the experimental conditions. To find the true dimensionality of the dynamics, we employed the delay embedding method (Takens 1981). The state of the system at time *t* was represented by a *d*-dimensional vector

(2) \begin{equation*} \mathbf{F}(t)=\left[f(t),f\left(t+\tau \right),f\left(t+2\tau \right),\dots f\left(t+\left(d-1\right)\tau \right)\right]. \end{equation*}

Here, *f(t)* is the field potential. The delay parameter τ is taken equal to the empirical autocorrelation time of *f*(*t*) (Pritchard and Duke 1995). The embedding dimensionality was 20, i.e. *d* = 20, judged enough to capture the underlying dynamics. This method is standard and has a long history in nonlinear system dynamics. The delay-embedded vector signal captures all generic information about the dynamics (Takens 1981; Pritchard and Duke 1995). We take this as a working hypothesis here as well.

From each *f(t)*, 20 delayed embedded versions were generated, with delays, τ = 59.9 ms. We used the common practise to choose τ (Pritchard and Duke 1995)*.* The delay parameter τ was chosen as the mean of 12949 original field potential time series as the value where the autocorrelation function of these time series = 1/*e* The distribution of τ is shown in Fig. S1. For each condition, the first 60 s of the *f*(*t)* was chosen as the basis of the embeddings.

### Dimensionality of state space

The main point of the data analysis was to preserve the field potentials as close as possible to their true dimensionality*.* The dimensionality of the dynamics was estimated with the Grassberger and Procaccia (1983) method by an automatic algorithm developed in-house. Specifically, for each point of the embedded time series, the distances to all other points in the state space were calculated. The number of points within a radius of ε was then counted for different values of ε. The correlation integral is given as

(3) \begin{equation*} {C}_{\varepsilon }=\frac{1}{n\left(n-1\right)}\sum_{\begin{array}{c}i,j=1\\{}i\ne j\end{array}}^n\mathrm{H}\left(\varepsilon -{\left\Vert{x}_j-{x}_i\right\Vert}_2\right), \end{equation*}

where $n$ is the number of points, ${x}_i$ and ${x}_j$ are $d$-dimensional coordinate vectors, $\varepsilon$ is the radius threshold, and $H$ is the Heaviside step function. The ∣∣ indicates the norm. The calculation of ${C}_{\varepsilon }$ is computationally intensive but parallelizing the code made it possible to reduce the computation time using the Danish Supercomputer center Computerome (http://www.computerome.dtu.dk). The algorithm arrives at the state space dimension by considering the dependence between the correlation integral and the distance thresholds for a given embedding dimension. Specifically, the slope of each curve produced by a log(*C*_E_) and log(ε) plot is automatically examined to determine the correlation dimension of the upper linear part of the curve (Fig. S1). The algorithm stopped for 48 embedded 60 s files because the estimated slope was out of range. The reason was the curve log(*C*_E_) versus log(ε) had a “knee” and included the part of the curve below the knee where log(*C*_E_) versus log(ε) was based on few data points. This is a known problem in experimental data (Babloyantz and Destexhe 1986; Roschke and Basar 1990; Pritchard and Duke 1995). In these 48 cases, the scientist via the code defined the start of the curve from which the slope should be calculated*.*

Finally, to determine the dimension, the dependence between the correlation dimension and embedding dimension is considered. If the correlation dimension is constant for 3 or more successive embedding dimensions, the algorithm concludes that the associated correlation dimension is the states dimension, in short correlation dimensionality (Fig. S1). The constant part of this dependence is determined by a least squares approach (Fig. S1). For 28 records, it was not possible to find a horizontal segment.

The correlation integral estimate depends on the number of sample points, here 30720 (60 s). Increasing the number of samples will increase the dimensionality slightly (Fig. S1) but further decreases the likelihood that the dynamics, and hence the dimensionality, remains (quasi) stationary. Therefore, the 60 s is a compromise giving an overall estimate of the dimensionality.

The correlation integral algorithm also determined the amount of false nearest neighbors, and the inequality *R*_*d* + 1_(*n*)/*R*_A_ > *A*_tol_ (see Kennel et al. 1992 for all details). If the embedding dimension chosen, in our case 20, is too low, the trajectories will intersect and overlap in state space. Such intersections and overlaps are called false nearest neighbors. False nearest neighbors thus tell that the embedding dimension chosen to reveal the dynamics is too low. *R*_A_ is an estimate of the size of the set containing all trajectories, *R*_*d* + 1_(*n*) the sum of distances between each of the *n* points and neighbors, and the *A*_tol_ set to = 1 (Kennel et al. 1992). When the number of dimensions increases the distances between the points in state space increase; this is especially true for time series with a limited number of samples. The above inequality limits the possibility that false neighbors at high dimensionality become true neighbors. If the data are contaminated with noise, it may be practically impossible find a dimensionality free of false nearest neighbors.

In each of the 3476 data epochs, we varied the *R_d_* systematically step by step to 21 dimensions as in Fig. S1B (left). In all epochs, the used correlation dimension was larger than the last false nearest neighbor. At embedding dimension 4, the number of false nearest neighbors was practically zero and the *A*_tol_ criterion fulfilled up to embedding dimension 20 (Fig. S1)*.* This means that the ***F***(*t*) unfolds as expected from a dynamical system at embedding dimension above 4 such that there are no crossings (intersections) of the trajectories (Kennel et al. 1992). For comparison, we shuffled the original time series to produced surrogate data with the random method (Matlab randperm) (Fig. S1).

In a comparison, the correlation integral dimensionality estimate seems superior to the alternatives of estimating dimensionality (Camastra 2003)*.* The extraction of the dynamics from the embedded times series of the field potential was done by using integer dimensionality close to the estimated correlation dimensionality. With the absence of false nearest neighbors and the *A*_tol_ criterion fulfilled, these factors together should give a close to exhaustive description of the dynamics contained in the field potential. The correlation dimensionalities are only rough estimates of the average field potential dimensionalities over 60 s.

### Dynamics confined to one part of state space

**Fig. 2:**
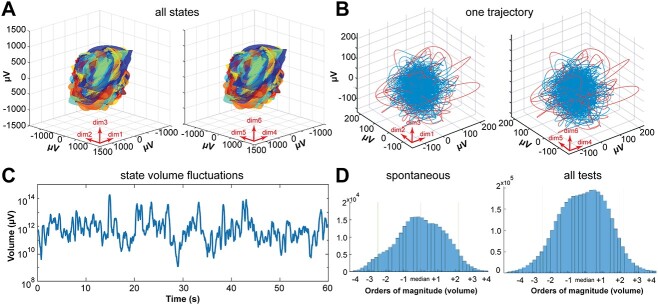
Each local cortical network constrains dynamics to one location. Here, the states evolve as trajectories whose volumes fluctuate. A) State space projections, dimensions 1, 2, 3 left, dimensions 4, 5, 6 right, containing all normal states in all patients and conditions, 3476 enclosing volumes (one color for each electrode lead-condition-patient combination. Notice, only the surfaces of the largest volumes are visible and projections of hyperellipsoids of dimensions 7, 8, and 9 are not shown; Methods). B) Each enclosing volume occupies a single, limited part of state space in shape of a hyperellipsoid. Inside each enclosing hyperellipsoid, the states over one 60 s epoch from lead 24 of patient 5 judging facial emotions make one trajectory. In this case, the state space and hence the trajectory is 6-dimensional. This 6-dimesinal trajectory is shown in 2 projections of the 6-dimensional state space. The two 3-dimensional projections show the dimensions indicated by the red arrows. Recording from cortex lining the posterior part of the superior temporal sulcus. Red: Superficial parts of trajectory. Blue: Part of trajectory densely occupying state space. C) Fluctuations of local state volumes from one lead (one local network). Each point represents the volume of 25 successive states, calculated by equation (4) (Methods). One epoch of 60 s of rest. Note log_10_ scale on the *y*-axis. D) Distribution of all local state volumes (equation (4)). *X*-axis log_10_*V* (orders of magnitude) aligned to the median. Left: Patient 12 during 20 randomly selected 60 s epochs of awake spontaneous activities. Right: All volumes in awake patients (*n* = 3363 epochs, each with 1200 log_10_*V* values).

Figure 2A shows all 106782720 normal states in one state space. These states were distributed in the 3476 individual sets. The set of points occupying state space were always multidimensional. The convex hull was computed for all relevant embedding integer dimensions. This was done using the Matlab function convhulln using tetrahedra forms as facets. Each of the 3476 sets including all states was covered in such an individual convex hull. The figure shows the smallest convex sets of 3476 that includes all 106782720 states. For the illustration, each of the 3476 sets was assigned one color; dimensions 1–3 and 4–6 were plotted separately in the figure. Also note that maximally 6 projections of this total state space are shown, i.e. projections of the seventh, eighth, and ninth dimensions containing relatively few sets are missing (Fig. 2A).

### Fluctuating dynamics

With data sampled over 60 s, the estimated dimensionality and the individual multidimensions of the state spaces are poor measures of how the dynamics evolve in the individual state spaces. The state space dimension is unable to reveal fast dynamics relevant for perception, thinking, and preparation and execution of behavior. To examine faster changes in state space occupancy, we approximated location of the states as a hyperellipsoid (see Fig. 2B). Mahalanobis distance is a measure of distances between data points in a multidimensional space, that is scale invariant and independent of Gaussian assumptions. Each state has a Mahalanobis distance in its multidimensional state space. These distances can be obtained from the center of the hyperellipsoid of the same dimension. All states from each of the 3476 60 s sets could be enclosed by 3476 hyperellipsoids. From each of these hyperellipsoids, we calculated the volume of the states of each of the embedded time series ***F***(*t*).

We also used the *embedded time series **F***(*t*) in their appropriate integer dimension to estimate the volume of the attracting set of states with a method detecting fast changes in state volumes. This method estimated the volume *V* of states accumulated over 50 ms. If the dimensionality of the individual state space was 5, we rotated the 5-diemnsional state space to have the maximal variance along the first principal component. In this case, the estimated hypervolume of the set of states in 5-dimensional state space was calculated as

**Fig. 3:**
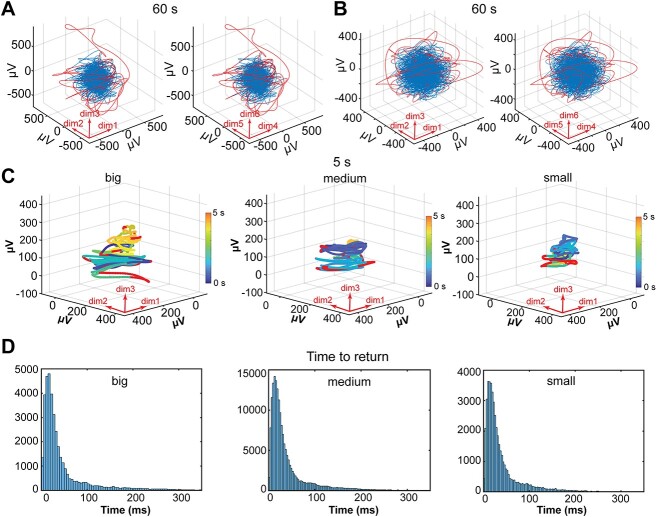
Trajectory behaviors. A) Projections of 6-dimensional state space of one 60 s trajectory, parahippocampal lead, classification of nouns. Blue part contains 90% of states making the dense part. Red parts contain 10% of states temporarily escaping the dense part. B as A, but lead in frontal opercular cortex, memory test 10, C) projections of first 3 dimensions of 5 s trajectories from the parietal operculum during classifying nouns, 3 example epochs of stretching and contracting trajectory of states making a big, medium, and small volume of states. Red parts: Outside sections of trajectory exceeding Mahalanobis distances at the 90% percentile from the center of gravity (Methods). The trajectories evolve according to the color-scale starting in dark blue, continuing as blue green and ending as orange. D) Distributions (log normal, see Fig. S4) of times to return to the dense 90% of states for outside (red) trajectories (*y*-axes) when the volumes are big, medium, and small.

(4) \begin{equation*} V=\sqrt{\operatorname{var}}1\ \sqrt{\operatorname{var}2\ }\sqrt{\operatorname{var}3}\ \sqrt{\operatorname{var}4}\ \sqrt{\operatorname{var}5}, \end{equation*}

in which the product of the square-roots of the variances along each of the 5 orthogonal principal components. This is a hyperrectangle giving the estimate *V* of the set in state space. The algorithm determined the *V* every 50 ms with a certain sliding window (was set to 128 samples = 250 ms). The 50 ms are less than τ (60 ms). The sliding window was less than 5 τ, because most data ranged between 5 and 7 dimensions (see Flow of states below). This means that each value of *V* is an estimate of the volume of 25 consecutive states. Note that this method estimates *V* as the volume of an orthotope (hyperrectangle) and not of a hyperellipsoid. In equation (4), the orthotope is 5 dimensional. If the individual state space was 7-dimernsional, the hyperrectangle volume was the product of the square-roots of the variances along each of the 7 orthogonal principal components. The succession of the 128 states was confined to a trajectory that however could take various shapes (as also seen for the 5-s trajectories in Fig. 3). The states, however over some 10–20 s become well bounded by a hyperellipsoid as the convex hull. *V* fluctuated roughly from 10^7^ to 10^17^ μV.

The volume expansions and contractions of local cortical states in state space were estimated in successive 50-ms intervals. Since we used only 25 states for the calculation of the volume, it made no sense to narrow the observation intervals. This does not preclude studies of faster fluctuations of the expansions and contractions. For example, if the sampling rate of the data is sufficiently high and if the autocorrelation in the time series is rapidly declining. The volume of the states varied significantly with the dimension of state space. Most likely, the dimensionality of the dynamics changes faster than over 60 s, the time needed to get a reasonably accurate estimate. However, the volume of the cortical states reflects smaller changes in dimensionality. If the distances between states increase, the product of the square roots of variances and hence the volume of the states increases (equation (4). This is roughly equivalent with an increase in dimensionality. Similarly, if the distances between states decrease, the variances and hence the volume of the states decrease. With our data, we could only observe volume fluctuations slower than the average flow of states. However, the flow of the states did not seem to fluctuate faster than the volume (see Fig. S6 values).

The amplitude variations of the fluctuations of the log_10_  *V* from the median in all 60 s test episodes in all awake conditions (*n* = 3526) in all patients (*n* = 13) were measured every 50 ms and shown in Fig. 2D. For comparison, the similar fluctuations from 20 randomly selected 60 s episodes in awake conditions, i.e. spontaneous behavior not belonging to any test episode, were also calculated (Fig. 2D). A coefficient of variation of the *z*-score values of *V* (see below) was also calculated in steps of 50 ms with a sliding window of 250 ms (Fig. S5).

### Putative attractor properties and behavior of trajectories in state space

The set of states as points occupying state space were located to one part of state space. Neither the states themselves nor the trajectories they formed did spread to parts of state space outside the convex hull in Fig. 2A. The reason for this could be that the network in which the states evolved had attracting properties, i.e. attracted states nearby. If we defined the hyperellipsoid as being within 90% limits of the Mahalanobis distances, the 10% external states (outliers) should be part of trajectories that sooner or later end up in this hyperellipsoid if the local network had attracting forces. We used the Matlab function mahal. The Mahal_Threshold_ = 90% percentile of Mahalanobis distances of all states from the center of the hyperellipsoid. The states included in the hyperellipsoid <Mahal_Treshold._

Figure S4G shows the probability densities of all states. We defined the putative attractor as the enclosed volume *V*_90%_ containing 90% of all Mahalanobis distances. Thus, 10% were outliers by definition.

Since the hypervolume, *V*, itself fluctuated over a scale from 0.2 to 10 Hz, during which *V* ranged from small to maximal, the attracting property should be universal, present when *V* was small, medium, and large. As the distribution of fluctuations in log_10_*V* was near Gaussian (Fig. 2D), we defined *small logV_90%_* as the lower 16% of the distribution of the 90% Mahalanobis distances in each patient, *medium* as the mid 68% of the distribution of log*V*_90%_, and *large* as the top 16% of the distribution of log*V*_90%_.

The algorithm then located small medium and big states of log*V*_90%_ and listed the length of the outlying states, i.e. the time in ms from departure to the return to the hyperellipsoid. This was done for all conditions and electrode leads (*n* = 3476) (Fig. 3C and D).

#### Flow of states

The ***F***(*t*) was visualized in the integer dimension just lower than the correlation dimensionality. If the correlation dimensionality was 6.6, for example, we chose 6 dimensions for visualization). Six dimensions gives 6 orthogonal planes (Fig. S4). The planes were defined by their normal vector and origin. The set contains only one, in this case, 6-dimensional trajectory, describing the evolution of states. Note that each state is identified in state space as, in this example, a 6-dimensional coordinate [μV*_ti_*, μV_*ti* + 1_τ, μV_*ti +* 2_*τ*, μV_*ti +* 3_*τ*, μV_*ti +* 4_*τ*, and μV_*ti +* 5_*τ*]. The states in state space progress every 1/512 s from one 6-dimensional μV value to the next. The 6-dimensional μV value however is composed from 6 samples picked in order with 60-ms interval. This is how the flow of states must be interpreted. If the Euclidian distances between successive n-dimensional μV values increase, the flow of states increases. When Euclidian distances between successive n-dimensional μV values decrease, the flow of states decreases.

To further prove the presence of attracting forces, we calculated flow vectors (Forsberg et al. 2016) as the average velocity over 4 samples (7.81 ms). Figure S4 shows the flow vectors after the time point where the Mahalanobis distance was maximal. We also calculated the Euclidian distance in μV between the point, where they exceeded 95% limit and the point of return to the enclosing volume in state space for all 3476 files (Fig. S4).

A detailed description of how the trajectories behave can be obtained from calculations of how long time it takes for states to cross one of the symmetry planes (Fig. S4). As an example, depending on where in this multidimensional space a continuous set of states are, they may first cross for example the third symmetry plane (from one side or the other), after a while they may cross the first symmetry plane and after yet a while the fifth and so on. The cross time is the number of states positioned on one side of the plane until they cross that plane (Fig. S4C, E and Fig. S6). Cross times were calculated for the whole material of 60 s files (*n* = 3476) (Fig. S6).

### Single trial analysis of the fluctuations

For the conditions with visual stimuli (visual tests 4, 5, 6, 11; emotions), the start of the stimulus is shown in Fig. 6. For the conditions in which the patients were verbally prompted to recall faces and places (memory retrieval tests 1, 2) and the classification of nouns, the trials are marked at the peak in the analog soundtrack (sampled with 30 KHz). For the visual stimuli conditions, the single trials were aligned to the start of the stimulus from the photodiode analog track (sampled with 30 KHz). An epoch from −4000 to +4000 ms was chosen. For each of these epochs, the log*V* signal was converted to *z*-scores by the Matlab function *z*-score. Using the peak in the soundtrack (providing the most well-defined trigger signal) to align all single trials in the 60 s episodes, segments, 8000 ms long, of *z*-scored *V* were calculated. Thereafter, all 8000-ms segments, one for each trial, were averaged and the average *V* plotted (Figs. 5 and 6). For the conditions in which the patients were verbally prompted to recall faces and places (memory retrieval tests 1,2) and the classification of nouns, the single trials were aligned to the peak in the soundtrack and similarly treated. This was done for each lead.

For each behavioral condition, the cross correlation between all leads of their individual average z-score was calculated by the Matlab function xcorr using the option coef, for the time interval −1000–+2000 ms. Thereafter, the pairwise lag between any two leads was calculated. A 95% confidence interval of the pairwise cross correlations served to separate significant pairs from random fluctuations.

### Anatomical registration

Image registration was applied for each patient’s images in order to compare all the subjects in a unique reference space. The applied procedure is depicted in Fig. S8. The MNI152 atlas is used as the global reference standard brain (Fonov et al. 2009). Each individual MRI image with all electrodes leads marked was transformed into standard format and merged with the MRIs in standard format of the other patients.

## Results

We recorded field potentials from multiple sites in the cerebral cortex of 13 patients with medical intractable epilepsy (Methods). In a local cortical network, the field potential is dominated by transmembrane currents from more than 10^5^ dendrites and their subsequent extracellular currents (Einevoll et al. 2014; Pesaran et al. 2018; Methods). Each field potential time-series reflects the underlying dynamics of the local network in the immediate neighborhood of the electrode lead at which it is recorded. All epileptic activity was removed. The analysis was restricted to those leads and time series that remained without any abnormality throughout the experiments. This left 3476 files of normal field potential recordings distributed over 290 electrode leads (Table 1, Methods) under 40 different conditions. Each file contains one epoch from one experimental condition from one electrode lead in one patient. The conditions comprised visual, auditory, gustatory, olfactory, and somatosensory stimulations, as well as tests with voluntary movements. We also included memory retrieval, mental navigation, and imagery tasks that did not involve external stimuli and overt behavior (Methods).

### Dimensionalities of the field potentials

The dimensionality is the number of dimensions we need to get an exhaustive account of the information about cortical states in the field potential (Roschke and Basar 1990; Stam 1996). The field potential recording is a time series, i.e. a 2-dimensional set of points varying in amplitude over time. However, theoretically, the field potential must be multidimensional because at the mm^3^ scale, it is produced by many transmembrane currents from many different neurons. Although the field potential is multidimensional, its true dimensionality is unknown. We therefore need first to determine the dimensionality of each of the 3476 field potential recordings. Once each of these field potential dimensionalities are found, we can create a multidimensional state space of that dimension for each recording. In this state space, one can observe the full multidimensional dynamics of each field potential showing how the cortical states produced by each local network evolve.

First, we embedded the field potential (Takens 1981) (Fig. 1 and Fig. S1; Methods). Then, we estimated the correlation dimensionality of the embedded field potential. Despite the intrinsic high dimensionality of the sources of the field potential, the estimated dimensionality was always comparatively low, ranging from 3 to 9 (Figs S2 and S3).

The individually estimated dimensionality for each condition and electrode lead was used to build a state space of that dimensionality, in which we studied the cortical states evolving from each local cortical network (Fig. 1; Methods). We used *Taken’s theorem,* stating that the delayed time-embeddings of a time series at its true dimensionality is geometrically equivalent with the dynamics generating the original time series (Takens 1981). The objective was to keep the data analysis from each individual epoch as close as possible to their estimated dimensionality. For each of the 3476 60 s experimental epochs, we then constructed individual state spaces. The dimension of an individual state space is the integer value of its fractal dimensionality. For example, if the individually estimated correlation dimensionality of one epoch was 7.1, or any fractal up to 7.9, the individual state space dimension would be 7. (The reason for this approximation is that the data do not exist in dimension 8). The dimensions of these 3476 individual state spaces ranged from 3 to 9. In each of these 3476 individual state spaces, we studied the cortical states evolving from each local cortical network (Fig. 1; Methods).

We can then reveal how a local cortical network contributes to cortical operations by changing its cortical states in a state space of true dimensionality. A local cortical state is one point in this multidimensional state space. In the state spaces in this study, one point represents the state of the local network over 1.95 ms (Fig. 1).

### Cortical states are confined to one part of state space

Figure 2A shows how all local cortical networks confine their evolving cortical states to a single region in state space. All cortical states in all patients (3476 60 s epochs, distributed over 290 leads, giving a total of 106782720 states) were located inside the enclosing volume of the multicolored hyperellipsoid (Fig. 2A). We found qualitatively identical, albeit smaller, hyperellipsoids at all electrodes leads and under all experimental conditions (Methods). Inside each hyperellipsoid, the temporal succession of states formed a trajectory (Fig. 2B and Fig. S4). Thus importantly, the dynamics even over longer time scales, 60 s, were confined to one single hyperelliptic region of state space.

In the figures, maximally 6 projections of the state spaces are shown when the state space was of dimensions 7–9. These projections may look similar, but they are not identical (Figs. S4J and S6), which is also apparent by a careful examination.

Data for 60 s or more are necessary to compute a reliable overall estimate of the correlation dimensionality (Methods), while the field potential dynamic cannot be regarded as stationary for so long. Consequently, the correlation dimensionalities are only rough estimates of the average dimensionalities over 60 s.

There is then a need for evaluating the dynamics at shorter, physiologically, and behaviorally relevant, timescales. In the rest of this study, we evaluate the dynamics inside these hyperellipsoids at physiologically relevant, ms, timescales. In state space, the states of a local network progress every 1.95 ms from one point with *d* coordinates in μV to the next (*d* is the dimensionality of the state space) (Fig. 2B).

### Fluctuations of the evolving volumes of cortical states

We rotated all state spaces of the evolving cortical states such that their variance was maximal along the first principal component. Using the variances, we estimated the volume of successive local states accumulated over 50 ms with a sliding time window of 250 ms in these 3476 individual data sets with preserved dimensions and shapes of trajectories (for choice of parameters, see Methods). The cortical states in such short time intervals form short trajectories, which are less well approximated by hyperellipsoids (Fig. 1). Therefore, each volume was calculated as the enclosing volume of a hyperrectangle (Methods, equation (4)).

Note that we use two complementary methods to estimate successive local state volumes. The hyperellipsoid method was used for successions of cortical states longer than 1 s (Fig. 2A, B). The hyperrectangle method was used for shorter successions of cortical states (equation (4), Methods; Fig. 2C and D).

The local networks generated states whose enclosing volume continuously expanded and contracted irregularly at every cortical site. For example, the networks generated states whose enclosing hyperrectangle volume continuously expanded and contracted during ongoing activity only (rest condition) (Fig. 2C) and during all (*n* = 110) measurements of the rest condition. All cortical networks generated these irregular fluctuating expansions and contractions under all tests and conditions (*n* = 3476 out of 3476) (Fig. 2D and Fig. S5; database). For comparison, we also plotted the similar volume fluctuations from 20 randomly selected 60s episodes during spontaneous behavior of one awake patient, i.e. in addition to the experimental conditions. In all cases, the ranges of the enclosing volume (from minimum to maximum) of the local cortical network states were at least four orders of magnitude, with no difference in the ranges between spontaneous behavior and experimental conditions (Fig. 2D right and left). Fluctuations of cortical field potential time-series are well known (Einevoll et al. 2014; Pesaran et al. 2018). Please note that it is not possible to infer from the 2-dimensional original field potential fluctuations that fluctuations would be the dominant feature of the evolving local cortical states in their true 5–9 dimensional state spaces (see also later results).

**Fig. 4:**
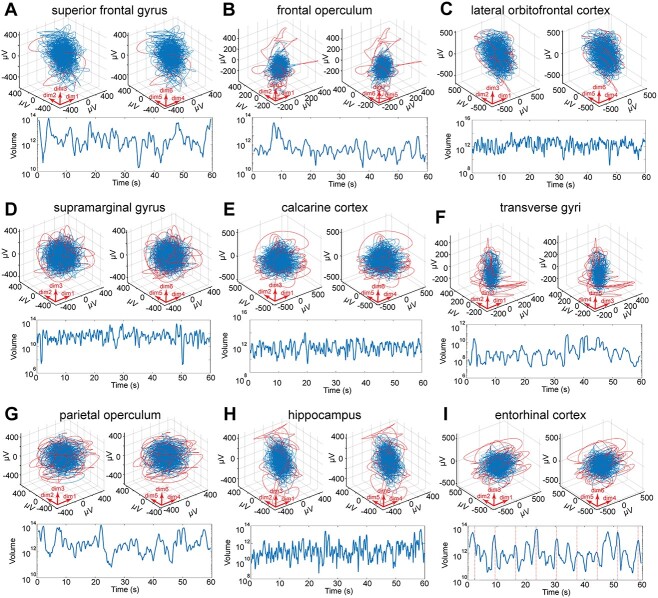
Different cortical network architectures create the same dynamic. Projections of trajectories over 60 s; red curves: trajectories temporarily exploiting nearby state space. Blue curves: trajectories inside the state space defined by the enclosing hyper-elliptic volume *V*_90%_. Below: fluctuations of the volumes of cortical states (equation (4), Methods) during the same 60 s. Time zero when the patient starts to execute the test. A) Stereognosis (patient 6). B) Conversation (patient 4). C) Ortho-nasal smell (patient 16). D) Motor test 6 (patient 17). E) Visual recognition (patient 17). F) Memory retrieval, mental navigation (patient 5). G) Watching TV (patient 12). H) Eating (patient 14). I) Facial emotion identification (patient 4).

Thus, inside each enclosing hyperellipsoid, the successive volumes of states fluctuated, often three orders of magnitude or more (Fig. 2D). The flow of states can be localized to well-defined trajectories, as shown in the projections. The volume fluctuations were irregular (Fig. S5; Supporting data repository) meaning that in each cortical network, shorter successions of cortical states expanded and contracted irregularly. Equivalently, one can describe the expansion and contraction as stretching and contraction of the multidimensional trajectory forming the succession of states (Fig. 1). These expansions and contractions continued under all conditions, with no exceptions (3476 out of *n* = 3476) (Supporting data repository) (for details and flow of states, see Methods).

### Attractor properties of the local network dynamic?

Fast volume fluctuations of the evolving cortical states of four orders of magnitude indicate that all local networks have unstable dynamics. However, the result that the states do not explore the whole state space but are confined to only one part of state space is a characteristic known from attractors (Babloyantz and Destexhe 1986; Roschke and Basar 1990; Stam 1996; Eliasmith 2007; Knierim and Zhang 2012; Rabinovich et al. 2012; Oprisan et al. 2015; Breakspear 2017; Strogatz 2018).

**Fig. 5:**
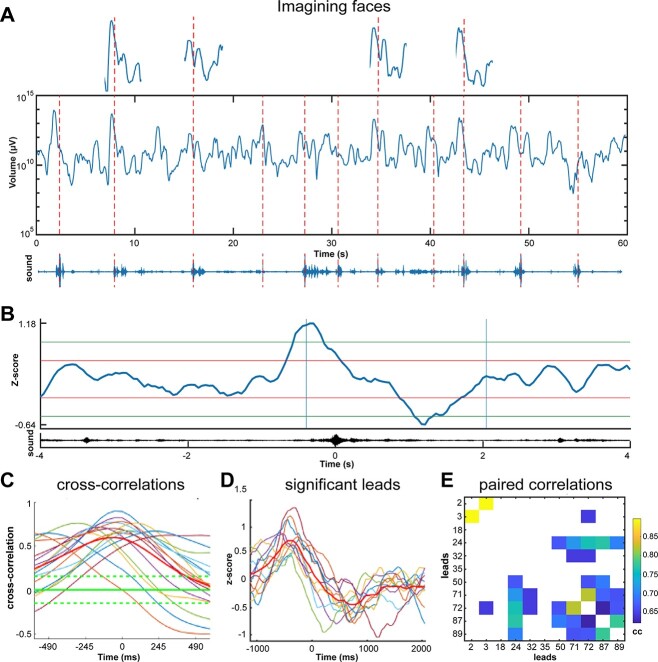
Single trial expansion and contraction. A) Fluctuations of *V* (*V* in equation (4)) in log_10_ scale, in 11 trials of recall of faces (memory retrieval test 1). Patient 17, lead 50, cortex in posterior part of the superior temporal sulcus. Above: 4 trials enlarged; below: Soundtrack with peaks indicated by the red stippled lines for each of the names of the 11 well-known persons to imagine (Methods). B) *Z*-score average of single trial volume changes; 4 s average of 11 *z*-scores showing one expansion and contraction. Average of the soundtrack below. Each trial was aligned to the peak amplitude of the sound. Vertical blue lines: The epoch displayed in C. horizontal red and green lines: 1 and 2 SD. The statistics determining significance was the pairwise cross-correlation between leads. C) Statistically significant cross-correlations of average *z*-scores between leads engaged in the task. Green stippled lines: 95% confidence limits. D) *z*-score averages of the volume expansions and contractions of the significant leads in C, with mean in red, note the different lags. E) Pairwise cross-correlations (cc) values of the leads in D.

**Fig. 6:**
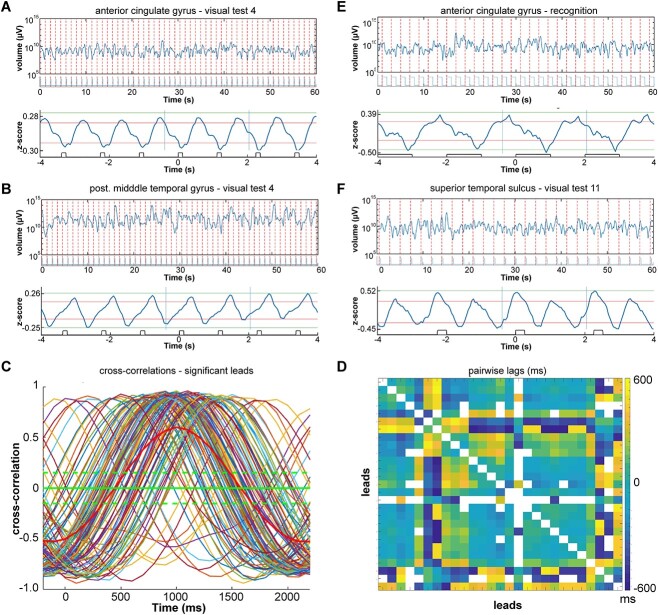
Entrainment of the expansions and contractions. A) 60 s epoch of fluctuating size of *V* (equation (4)) in log_10_ scale with onset of trials marked with red stippled lines. Bottom: average *z*-scores of volume fluctuations over trials with average stimulus presentation. Natural pictures presented 125 ms with 1-s black screen intermissions. Patient 11 lead 2, anterior part of cingulate gyrus. B) Same patient, same test, but lead 101; phase shift of the fluctuating expansions-contractions (compared with A). C) Pairwise significant cross-correlations of average *z*-scores above 70% quantile during the task for the time segment defined by the 2 vertical lines in A bottom. Green stippled lines: 95% confidence limits. Thick red curve: mean values. D) Matrix of the pairwise lags for all leads in C. E) Visual recognition test, patient 6, lead 2. Average of *z*-scores (top), 60 s episode of recognition of fluctuating size *V* in log10 scale with photodiode signal of picture presentation below. F) Fluctuating volume entrainment of double frequency. Geometrical pictures (visual test 11) patient 5, lead 25, cortex lining posterior part of superior temporal sulcus.

Figure 3A shows a typical trajectory inside one enclosing hyperellipsoid. For most of the time, the trajectory makes up a dense part of the hyperellipsoid. Near the surface, however, the trajectory (red) escapes the dense part (blue) for a while, for thereafter to return to the dense part. If we define the putative attractor as the (dense part) set of states whose Mahalanobis distances from the center of the enclosing hyperelliptic volume were less than 90% of the maximum measured value (Fig. 3A and B). First, we examined the fate of outside (red) trajectories of states at time intervals from 0.5 up to 60 s during all conditions and tests (*n* = 3476). Since the volume of the putative attractor continuously changed, the states outside the putative attractor should return, no matter if the putative attractor is small or large. Therefore, we examined the fate of outside trajectories of states when the enclosing volume of the putative attractor, because of its fluctuations, was small, medium, and large (for definitions see Methods). Thus, large volumes, medium-sized volumes, and small volumes all had 10% of states as (red) outliers (Fig. 3C). Irrespective of the size, all trajectories of states returned to the putative attractor (Fig. 3D). The point of return was at a different position than that from which they had departed (Fig. S4J).

Changing the definition of the outside states to 20 or 5% did not change the distribution of the return times (Fig. S4**;** see also Fig. S4, for examination of other attractor properties). In summary, we have shown that when the trajectories escape the dense volume for a while, they rapidly return to the blue, dense part at another position.

A requirement is that no subset of the attractor is an independent attractor or other dynamics. We examined the details of the flow of states in state space. The state flows during all 3476 tests and conditions were fast in all dimensions (Fig. S6). The state flows did not reveal any evidence for other localized attractors or other dynamics (Fig. S6). Thus, no subset of the putative attractors were independent attractors or other dynamics (Fig. 3C, Figs. S4 and S6). Although this requirement is fulfilled, the local network dynamic should also meet two other essential requirements: the attractor should occupy an invariant part of state space and have a basin of attraction (Strogatz 2018).

None of our experimental conditions succeeded to perturb the putative attractor systematically (Figs. 2A, 3D and Fig. S4). Therefore, we cannot prove that initial states trajectories outside the attractor candidate (in its basin of attraction) return to the dense part. To prove attractor dynamics, the demonstration of a basin of attraction is essential (Strogatz 2018). In addition, the trajectories in all 3476 epochs continuously expand and contract. The large fluctuations of contraction and expansion of the trajectories in state space are incompatible with the definition of attractors (Strogatz 2018).

Each local cortical network produces local cortical (postsynaptic) states. We have shown that succession of states is kept together in a trajectory occupying only one region of state space. Within this region, there are no other types of dynamic. Trajectories fluctuate, showing large contractions and expansions. Moreover, despite these fluctuations, states temporarily escaping the dense part are rapidly pulled back. This means that the local cortical dynamic we found has stability, but the dynamic also has properties which are not properties of known attractors. Especially, the expansions and contractions of the trajectories are incompatible with the definition of attractors (Strogatz 2018). We refer to this fluctuating expanding and contracting, but stable dynamic as the local cortical network (postsynaptic) dynamic.

### The local cortical network dynamic is a universal large-scale cortical state dynamic

In these 3476 epochs, comprising all tested conditions and all 290 leads we could not find any exception from this local cortical network dynamic (Figs. 2-4 and Figs. S4, S6; supporting files. https://drive.google.com/drive/folders/1Fi44M3Xt_HwkId21EWF3hSOh6Peb0WYt?usp=sharing).

This means that local networks from different cortical locations and within different cortical areas generated the same type of dynamic to drive their cortical states. This dynamic is different from that of Hopfield (1982) networks and other network models. Indeed, local cortical networks in prefrontal cortex, primary visual cortex, supramarginal gyrus, and hippocampus have widely different anatomical and neurotransmitter receptor architectures (Elston 2003; Zilles and Amunts 2010). Notwithstanding these architectonic differences, the local networks created cortical postsynaptic states using the same dynamic (Fig. 4; supporting data).

Similarly, cortical states evolved under this dynamic created by the local networks during all tested conditions. For example, cortical states during spontaneous behavior, rest condition, and during different brain functions such as mental navigation, classification of nouns, somatosensory attention, tasting sweets, etc., were all evolving under the same dynamic (Fig. S7; supporting files). These results show that all tested local networks in the cortex produced one type of dynamic, which prevailed in all tested conditions. However, from the results presented so far, one cannot tell whether the fluctuations signify task-related activity or activity unrelated to a task.

### Subsets of local networks cross-correlate their expansion and contractions

To pursue how the fluctuations might relate to the tasks patients were doing and pursue how local networks might interact during tasks, we focused on single trial tasks with multiple trials to get sufficient statistics.

In one of these tasks, when the experimenter spoke the name of well-known person, the patient should imagine the face of that person. When patients engaged in this task, some local networks increased their volumes of the evolving states often peaking at the start of the spoken name (Fig. 5). A slightly longer contraction/slowdown of the evolution of states ensued the initial increase/acceleration of the states, during the rest of the spoken name and the imagery (Fig. 5A and B). Similarly, other such single trial tasks in which patients were asked to identify facial emotional expressions, imagine well-known landmarks or tourist attractions (Fig. S5I) a subset of local networks generated a small expansion followed by a contraction. This was also the case when the patients judged whether spoken nouns were concrete or abstract (Fig. S5L), looked at geometrical shapes (Fig. 6F), and recognized natural scenes (Fig. 6E). Thus, a subset of local networks generated a small expansion followed by a contraction concurring with the imagery, problem solving and perception (Figs 5, 6, and Fig. S5). The contraction is equivalent with a compression of the trajectories in these single trial tests (Figs 1 and 3C). Each patient completed from 2 to 8 such single trial tests (Table S1). A particular subset of local networks generated a single expansion/acceleration followed by a contraction/slowdown of the evolving states for each of these single trial engagements.

But how do the cortical networks interact to produce this type of engagements? For engagement in the face imagery test, many local networks, but not all, generated similar singular expansions and contractions of evolving local cortical states (Fig. 5C and D). This subset of local networks showed a pairwise significant cross-correlated expansion and contraction of the local cortical states. These pairwise cross-correlations appeared with different lags (Fig. 5C and E). However, not all local cortical networks produced such significant cross-correlations. Only a subset of the many local networks cross-correlated their expansions and contractions of their local states during the single trials, while the remaining had no significantly cross-correlated fluctuations (Fig. S5).

These results also held for engagements in other tests with multiple single trials. A larger subset of the local networks cross-correlated their single expansion-contraction, always with different lags. During the contraction, the volumes of the evolving states often reduced to 1/10 or less of the mean volume (Figs. 2, 4, and 5). When the volumes contract in all members of the subset, these local networks slow down their evolutions of cortical states and confine the states to a smaller part, closer to the center of state space (equation (4), Methods).

With few exceptions, a particular combination of cross-correlated local cortical networks generated these test engagements (Table S1). On average, 43.5 ± 24.8% (SD) of the local networks cross-correlated evolutions of their cortical states in these tests (*n* = 43). Thus, the local networks interacted by cross-correlating their expanding-contracting cortical state evolutions. At the macroscopic scale, different, single trial tests engagements seem to be operated by different combinations of cortical local networks.

We also observed expansions and contractions, comparable to those in Fig. 5, in the absence of sensory stimulation. All expansions and contractions of state volumes and their relations to the conditions can be found in the Supporting data.

### Entrainment of cortical states

A look at the expansions and contractions of local cortical states in Figs 2, 4, and 5 emphasizes their irregularity (Fig. S5) as true fluctuations and the absence of oscillatory dynamics. For a long time, there has been a controversy between scientists who regard asynchronous irregular cortical states as characteristic for the cerebral cortex, and others who emphasize oscillations as fundamental for cortical operations (Einevoll et al. 2014; Dehghani et al. 2016; Breakspear 2017; Muller et al. 2018; Pesaran et al. 2018; Buzsaki 2019; Singer et al. 2019; McCormick et al. 2020).

However, oscillations may hide in the true multidimensional field potential. Local networks entrained their expansions and contractions of their evolving states during visual tests 4, 5, and 11. In these tasks, patients were instructed to watch the items shown on the monitor, without responding. A few, up to all, local networks monitored engaged in an entrainment of the expansions and contractions of the states, with a frequency identical to (Fig. 6A and E), or (occasionally) double that of the stimulus presentation rate (Fig. 6F). Rhythmic visual presentations with no intermission of a black screen (visual test 3) and stimulus durations of 6 s (facial emotions) did not produce entrainment. In contrast, regular presentation of visual stimuli of 125- or 1000-ms duration, at 0.5 or 1 Hz, did produce entrainment (including visual test 6, recognition).

As for the other single trial tests with multiple trials, each trial was associated with one expansion followed by one contraction (Fig. 6A, B, E) or occasionally two expansions and contractions (Fig. 6F). The phase of the expansion–contraction oscillation varied with cortical location. In some local networks, the expansion led the stimulus presentation (examples in Fig. 6A and E), while at other sites, it trailed it (Fig. 6B) or peaked at the stimulus presentation (Fig. 6F). The frequency of these entrained oscillations was strictly locked to the presentation frequency or the exact double of the presentation frequency (41 out of 41 test completions) (Fig. 6A, B, and F). The entrainment by the local networks always had lags ranging from −500 to +500 ms (like the example in Fig. 6C, and D).

The proportion of local networks showing entrainment in the four visual tests (4, 5, 6, and 11; Methods) ranged from 0 to 100%. From 91 to 100% of the local networks monitored (mean 97%; *n* = 8) entrained when natural pictures were presented for 125 ms at 1 Hz. The entrainment, with different lags, was evident only in the stimulus onset time-locked averaged evolution of the states (Fig. 6A, B, E, and F). This shows that the system is not simply undergoing oscillatory (limit-cycle) dynamics (Fig. S7). The time-locked averaged oscillations here are just another property of the multidimensional dynamic of states.

## Discussion

We extracted and identified the dynamics of postsynaptic states, contained in field potentials, with an unbiased method. From the results, we can conclude that each local cortical network at this mm^3^ scale generated the same type of dynamic. Thus, collective postsynaptic operations by cortical neurons in a local network are governed by one principle. All examined local cortical networks produced successions of local cortical states forming trajectories that expanded and contracted irregularly and unceasingly during internal, sensory, cognitive, and motor activities. Local networks, forming a subset of all networks, produced a single expansion and contraction associated with a particular single trial task. This set of single expansions and contractions cross-correlated among the members of the subset. Our data left no evidence of any stationarity, stable patterns, or equilibria or other alternative types of dynamics. This, hitherto unreported, fluctuating expanding and contracting, but stable dynamic gives a simple general explanation of larger scale (postsynaptic) dynamics of the cerebral cortex at behaviorally relevant timescales.

The local cortical networks postsynaptic dynamic was derived from the actual cortical field potential, instead of being a computational model or a theoretical attractor model borrowed from physics or other disciplines. By extracting the field potential dynamics and examine it in state spaces of sufficiently high dimensionalities (Takens 1981) and by avoiding methods requiring stationarity, linearity, and averaging, we revealed this dynamic.

A low-dimensional, slowly decaying, oscillating spiral attractor was recently described (Bruno et al. 2017). However, we were unable to find dynamics such as the local cortical networks in the literature. Since the local network dynamic is based on postsynaptic processing, it is also distinct from spiking dynamics, which typically unfold in low dimensional state space (Churchland et al. 2010; Forsberg et al. 2016; Gallego et al. 2017). Putative attractors of similar dimensionalities to those of the local cortical network dynamic, but derived from the human EEG, have been proposed (Babloyantz and Destexhe 1986; Roschke and Basar 1990; Stam 1996) based on a working assumption of stationarity. It is possible that these putative EEG attractors are in fact the local cortical network-type dynamic, which could be tested experimentally. We are not aware of any network, computational, or mathematical model that can produce the local cortical network dynamic.

Whereas our results may not support earlier models and interpretations, they are, to our knowledge, not in conflict with existing data. For example, both fluctuating states and entrained oscillatory cortical states can arise from the local cortical network dynamic. This may resolve the controversy between scientists who regard asynchronous irregular cortical states as characteristic for the cerebral cortex, and others who emphasize oscillations as fundamental for cortical operations (Einevoll et al. 2014; Dehghani et al. 2016; Breakspear 2017; Muller et al. 2018; Pesaran et al. 2018; Buzsaki 2019; Singer et al. 2019; McCormick et al. 2020).

This expanding and contracting dynamic is different from the dynamics of attractors known from complex dynamical systems or models (Hopfield 1982; Babloyantz and Destexhe 1986; Roschke and Basar 1990; Stam 1996; Eliasmith 2007; Tognioli and Scott Kelso 2011; Knierim and Zhang 2012; Rabinovich et al. 2012; Priesemann et al. 2013; Wimmer et al. 2014; Oprisan et al. 2015; Breakspear 2017; Strogatz 2018). If the local cortical network dynamic is not an attractor-type dynamic, then which forces keeps the states together? Why do not they escape in any of the 3476 epochs and explore other parts of state space? This stability might depend on the delayed balance between excitation and inhibition in the local networks.

### Evolving postsynaptic cortical states and local excitation and inhibition

The field potential is a weighted sum of the extracellular currents in the neighborhood of the electrode lead. The main sources of these extracellular currents are the underlying multiple postsynaptic membrane currents (Einevoll et al. 2014; Pesaran et al. 2018). The field potential is measured at a relatively coarse mm^3^ scale, causing many details of the biophysical computations of inward and outward currents to get lost (Iacaruso et al. 2017; McCormick et al. 2020). The field potential is closely correlated and in phase with the voltage sensitive dye signal in the cerebral cortex of rodents, carnivores, and awake primates (Arieli et al. 1995; Ferezou et al. 2007; Lippert et al. 2007; Ayzenshtat et al. 2010).

When excitation dominates the local cortical network, this gives a similar short stretching of trajectories in state space, followed by a slower 1s contraction when inhibition dominates (Roland et al. 2017). Thus, the single trial expansions and contractions of cortical states we find in human local networks may relate to local dominance of excitation, followed by local dominance of inhibition. Without excitation, states might not progress and expand in state space. Without inhibition, evolving states may not contract, and states escaping from the attractor might not be pulled back (Roland et al. 2017). The ongoing expansions and contractions in state space observed in the absence of experimental stimuli may also reflect such shifts.

### The local cortical network dynamic and its relations to architecture and function

Local networks in prefrontal, parietal, and temporal cortices, hippocampus, and amygdala all generated the same dynamic. These networks have well-described different anatomical and receptor architecture (Zilles and Amunts 2010). That local networks with widely different architectures, at the mm^3^ scale, operate with the same dynamic may seem counterintuitive. First, however, it may be an advantage that local networks with different architectures can maintain stability. Second, the different networks may need a common dynamic to perform large-scale operations co-operatively. If local networks operated with different dynamics, it would be challenging to adjust the dynamics mutually and swiftly. Similarly, our other main result that spontaneous activity and all the different brain tasks we examined emerged under the same dynamic may seem even more counterintuitive. However, having only one (type of) dynamic operating under all conditions may be an advantage. It means that the cortex does not need to change dynamics to react to new transients, to shift to another brain function or switch from internally initiated functions to externally initiated functions and vice versa. The result that a subset of cortical local networks cross-correlated the expansion and contraction of their evolving cortical states in all the single trials tests supports this statement.

The progress of states, their expansions, and contractions continued without external signals or demands. This result and the finding that all conditions evolved under one type of dynamic indicate that local cortical network dynamic is a continuous intrinsic large-scale dynamic of cortical local networks that the networks can modify, but not change or replace by another dynamic in response to external demands or stimuli. Local cortical network dynamic then is a general underlying dynamic created by local cortical networks that ensures physiological stability, sensibility to inputs, and mutual interactions.

### Cortical state space behavior and contribution to cortical function

Since the trajectories of the multidimensional field potential in state space continuously stretched and contracted under all conditions, our results are incompatible with dynamics temporarily settling into particular stable states (equilibria) or representations. This applied even to conditions in which patients perceived stationary pictures or imagined stationary objects.

In a single trial, a subset of local networks produced a short expansion, stretching the trajectory, followed by a 1–2 s continuously changing contraction of the trajectory. When the trajectory stretches, the sequence of evolving states (making the trajectory) gets stretched (evolve faster). When the trajectory contracts, the state-space–time shortens (evolve more slowly) and the states get more compact and their variance diminish (equation (4), Methods). The expansion may relate to the excitation of each of the networks in the subset. Locally, each network thereafter switches to an inhibition dominated regime increasing the contraction of the trajectory, as argued in the section above. The members of the subset of local networks then slow down their evolutions of states, reduce state variances, and confine the states to a smaller part, closer to the center of state space. This collective, but temporary, convergence of states at multiple cortical sites was coupled to a collective reduction in dimensionality due to the cross-correlation among the engaged local networks. We interpreted these mechanisms as signs of engagement in the experimental task, because the dimensionality reduction is more likely to signal the result of a collective operation, than the activity of the un-correlated local networks (Breakspear 2017; Roland et al. 2017). Our results also agree with recent data showing that fluctuations of the intracellular Ca^2+^ cross-correlate among cortical areas engaged in visual perception (Ebrahimi et al. 2022). Furthermore, the entrainments of the expansion and contraction (Fig. 6) indicate that the entrained networks are engaged and adjust their excitability, some of the entrained networks in anticipation of the next sensory event in accordance with earlier human studies (Besle et al. 2011; Gomez-Ramirez et al. 2011). This anticipatory expansion may also appear in the tasks with slower, but rhythmic presentations (Fig. 5, supporting data).

### Local cortical network dynamic and spatio-temporal cortical dynamics

Local networks in the cortex interact *spatially* to contribute to cortical functions (Arieli et al. 1995; Ferezou et al. 2007; Ayzenshtat et al. 2010; Iacaruso et al. 2017; Roland et al. 2017; Davis et al. 2020; Grün et al. 2022). Such mutual spatial interactions most likely also contribute to create the local network dynamic. However, a recording from a local electrode showing the collective postsynaptic response of the neurons in the local network is blind to the spatial interactions between neurons within the network. This means that finer biophysical details underlying perception, imagery, motor control, and other tasks, which we could not resolve at this mm^3^ scale, will have to be resolved by dense recordings locally (Ferezou et al. 2007; Harvey and Roland 2013; Dehghani et al. 2016; Grün et al. 2022).

The different lags of the expansion and contraction among the local networks in the single trial tasks are compatible with overall progressing larger scale spatio-temporal dynamic in cortical space (Roland et al. 2017; Muller et al. 2018; Davis et al. 2020; Grün et al. 2022). However, the depth-electrode lead sites were neither evenly nor densely distributed over the cortex but were sparsely scattered in individual patients. This precluded a sufficient coherent anatomical reconstruction of the lead positions for examining systematic relations between the spread and progress of the expansions and contractions over the cortex and execution of the spontaneous activity or brain task. Neither was this study designed to examine how local network state fluctuations distinguish different single percepts, imageries, thoughts, and behaviors.

### Perspective

All local cortical networks, at the mm^3^ scale, only create one type of postsynaptic dynamic. This dynamic is characterized by fluctuating expanding and contracting evolving states and stability. The networks do not change this dynamic under physiological conditions. This reinforces the notion of cortical autonomy. Instead of changing the dynamic, special subsets of local networks adapt and cross correlate the contraction of their evolving states, when they engage in a single trial task. These cross-correlations were concomitant with external inputs reaching the cortex, imagery, memory retrieval, and overt behavior. To what extent these temporary convergences of collective states relate to subjective percepts, imagery, or task solutions, are challenges for future studies. This study is just a start, revealing a general dynamic enabling cortical networks to switch between tasks and spontaneous activities. It is a multidimensional dynamic, generating irregular state fluctuations as well as entrained oscillations. Since our results are robust, they should be reproducible. The fluctuating expanding and contracting, but continuously stable dynamic is a mathematical object. This makes it an overall, simple description of how cortical states evolve, which can be tested experimentally.

## Supplementary Material

Supplem_Materials_Willumsen_20220917_tgac040
