# Supplementary material for: Local networks from different parts of the human cerebral cortex generate and share the same population dynamic
Source: Cereb Cortex Commun. 2022 Oct 28;3(4):tgac040. doi: 10.1093/texcom/tgac040 (PMC9753090; doi:10.1093/texcom/tgac040)
Supplement: Supplem_Materials_Willumsen_20220917_tgac040 [file supplem_materials_willumsen_20220917_tgac040.zip › Supplem_Materials_Willumsen_20220917_tgac040.pdf]

Supplementary Materials for

Local networks from different parts of the human cerebral cortex generate and share the same population dynamic

Alex Willumsen[1], Jens Midtgaard[1], Bo Jespersen[2], Christoffer K.K. Hansen[1], Salina N. Lam[1], Sabine Hansen[1], Ron Kupers[1,3], Martin E. Fabricius[4], Minna Litman[5], Lars Pinborg[5,6], José D.Tascón-Vidarte[3], Anne Sabers[5], Per E. Roland[1*]

This PDF file includes:

Figures. S1 to S8
Table S1

Correspondence to: perrol@sund.ku.dk

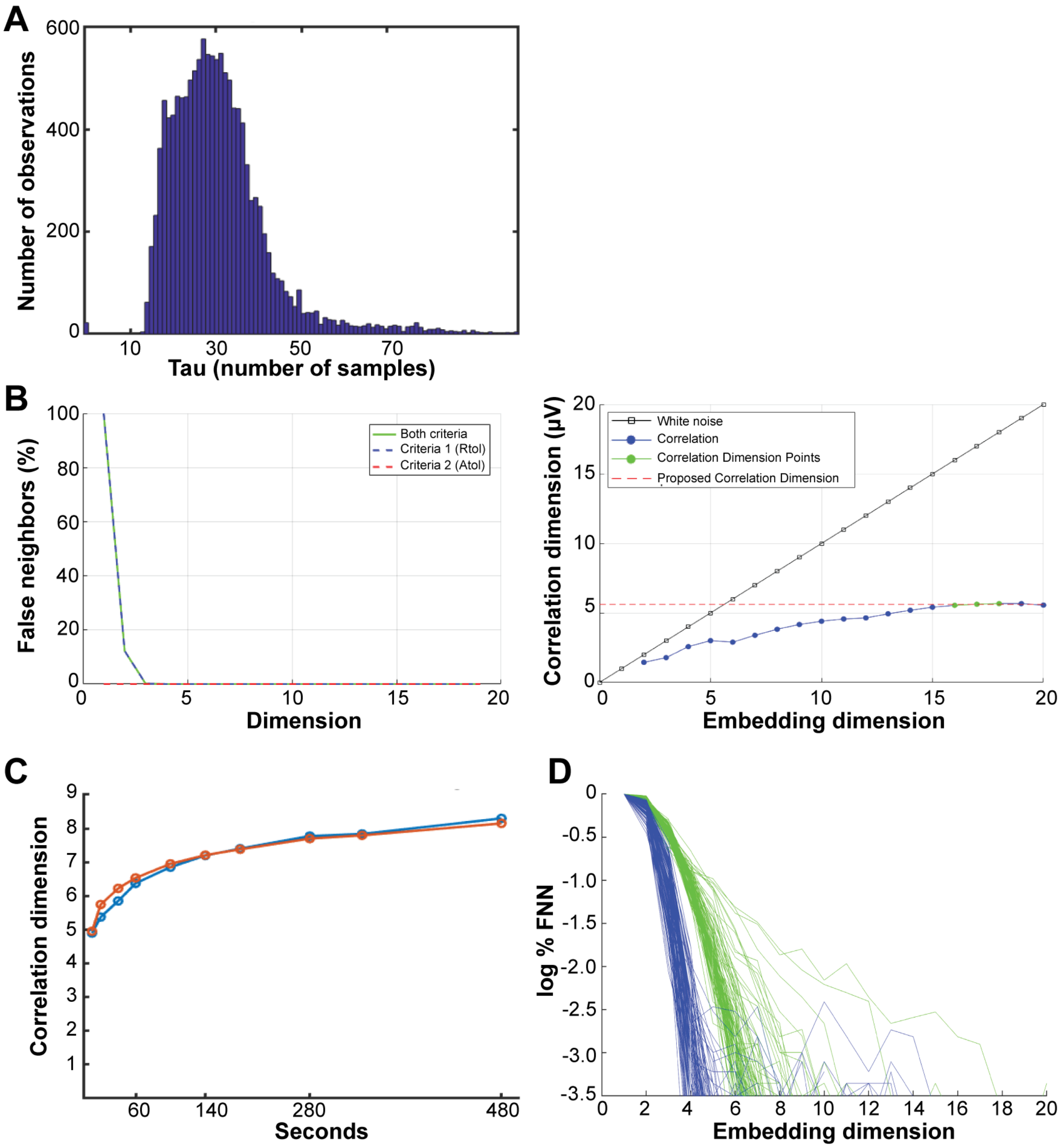

Fig. S1. Estimation of the dimensionality of *f*(t). A. Distribution of 12949 optimal values, chosen at the time when the autocorrelation function equals 1/e in the original f(t) time series. The τ mean = 30.65 samples; equivalent to 59.9 ms. All correlation integrals were calculated with τ = 30 samples. **B.** Algorithm for estimating the correlation dimension of the state space. Left: false nearest neighbors and the $R_{d+1}(n)/R_A$ criterion as function of number of embedding dimensions. Right: the algorithm finds the first horizontal segment of the curve relating embedding dimension to correlation dimension. **C.** Estimated correlation dimension as a function of data samples. Data from patient 4, lead 1 (blue) and lead 53 (red). With increasing number of samples, the correlation dimension continues to increase, presumably a consequence of decreasing stationarity. **D.** False nearest neighbors (FNN) in original (blue) and shuffled data (green).

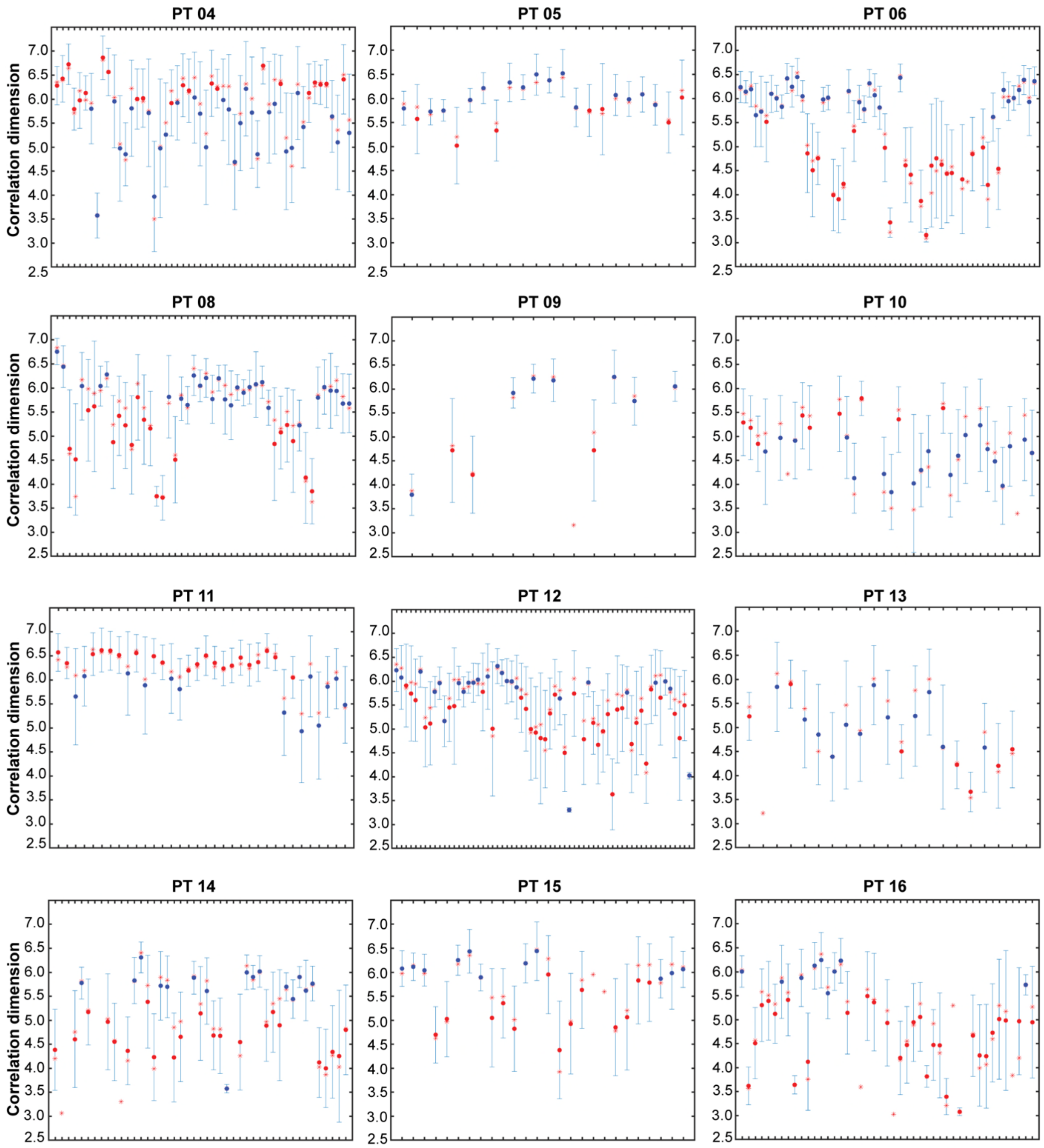

Fig. S2. Distributions of the mean correlation dimensionalities. Correlation dimensions for all tests completed by the patient along the y-axes; x-axes: positions of electrodes leads (not grouped anatomically). Red star: median. Red or blue dots: mean. The dot color depends on the membership of cluster (**Figure S3**). Standard deviation shown as error bars.

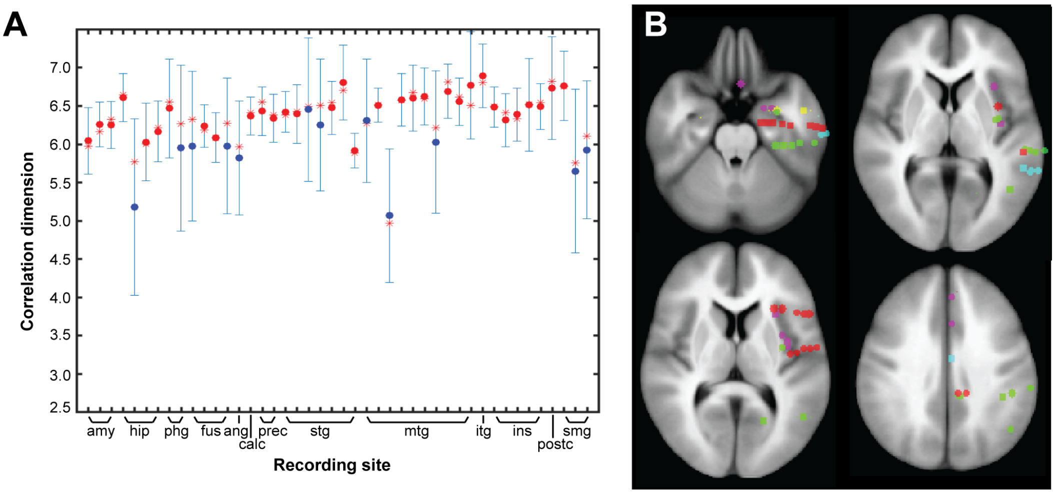

Fig S3. Correlation dimensionalities of field potentials in patient 17. A. Correlation dimensions as function of anatomical location for all tests of patient 17. Red star: median. Red or blue dots: mean, depending on membership of cluster. Standard deviation shown as error bars. Abbreviations: amy: amygdala; hip: hippocampus; phg: parahippocampal gyrus; fus: fusiform gyrus; ang: angular gyrus; calc: calcarine cortex; prec: precuneus; stg: superior temporal gyrus; mtg: middle temporal gyrus; itg: inferior temporal gyrus; ins: insula; postc: postcentral gyrus; smg: supramarginal gyrus. **B.** Axial slices of the standard brain, showing positions of electrode leads. Green spots for leads from patient 17; other colors refer to other patients.

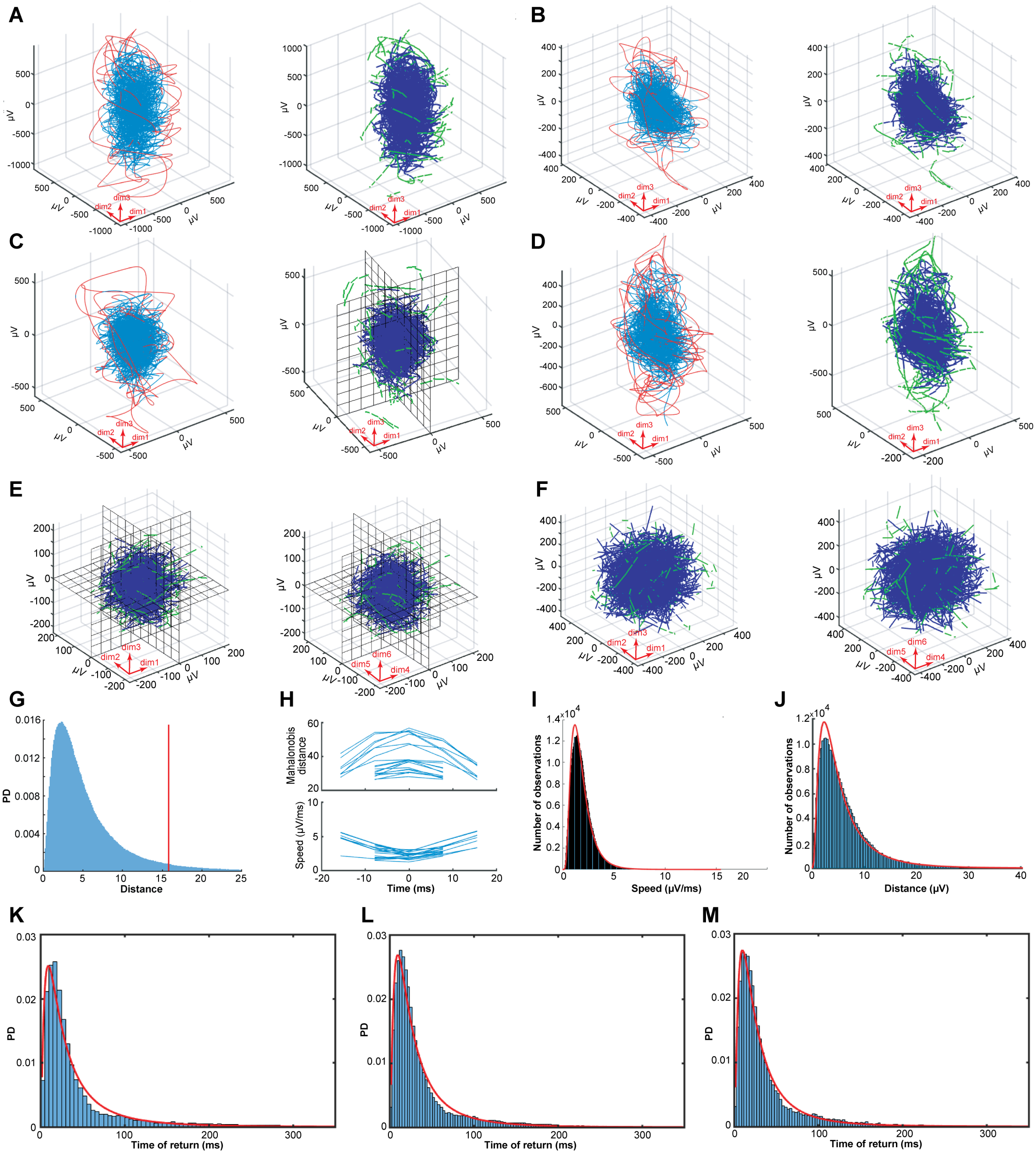

Fig. S4. Trajectories, flow and putative attractor properties. In **A** to **D,** the putative attractor is defined as the hyper-ellipsoid containing 95% of all states over 60 s. When the trajectory is dense, it is blue, when it is outside it is red. Only projections of the first 3 dimensions are shown. Panels are from 4 different patients. In A to D, trajectory and vector flows (**Methods**) are shown to the left and right, respectively**.** Vectors in the dense part are blue and vectors outside pointing towards the dense part are green. **A.** Slow wave sleep, Correlation dimensionality (CD): 2.85, anterior part of insula. **B.** Recall places, CD: 5.85, anterior part of cingulate gyrus. **C.** Motor sequence (motor test 6), CD: 6.05, mesial part and anterior part of superior frontal gyrus. Right: flow vectors with 2 symmetry planes (different angle of view chosen for clarity). **D.** Eating, CD: 5.81, amygdala. **E.** Flow vectors pointing towards the dense part in projections of 6 embedding dimensions with corresponding symmetry planes (as used in **Figure S6** for the calculation of flow of states). Judging the moods from facial expressions, lead 24, cortex lining posterior part of superior temporal sulcus. Left: first 3 dimensions; right: dimensions 4,5,6. **F.** Flow vectors,

first 60 s of mental navigation (test 8), lead 25, frontal eye field. **G.** Histogram of Mahalanobis distances from the center of the hyper-ellipsoids for all states in all 3476 combinations of leads-conditions with normal field potentials; y-axis: probability density (PD). Red vertical 95% limit. **H.** Representative distance and velocity profiles of departures from the dense part, aligned to maximal distance from the dense part (time = 0). lead 4, imagery of faces. **I.** Logarithmic-normal distribution (red curve) of speed of all flow vectors (n = 506,409) towards all dense parts. **J.** Log-normal distribution of distances (n = 253,490) between the point of departure and re-entry into the dense part (**Methods**). **K.** Log-normal probability density (red curve) of times to return to the dense part for 10% outlying states when the V is small; median 23 ms. **L.** Idem for medium size V, median 24 ms. **M.** Idem for large V, median 23 ms.

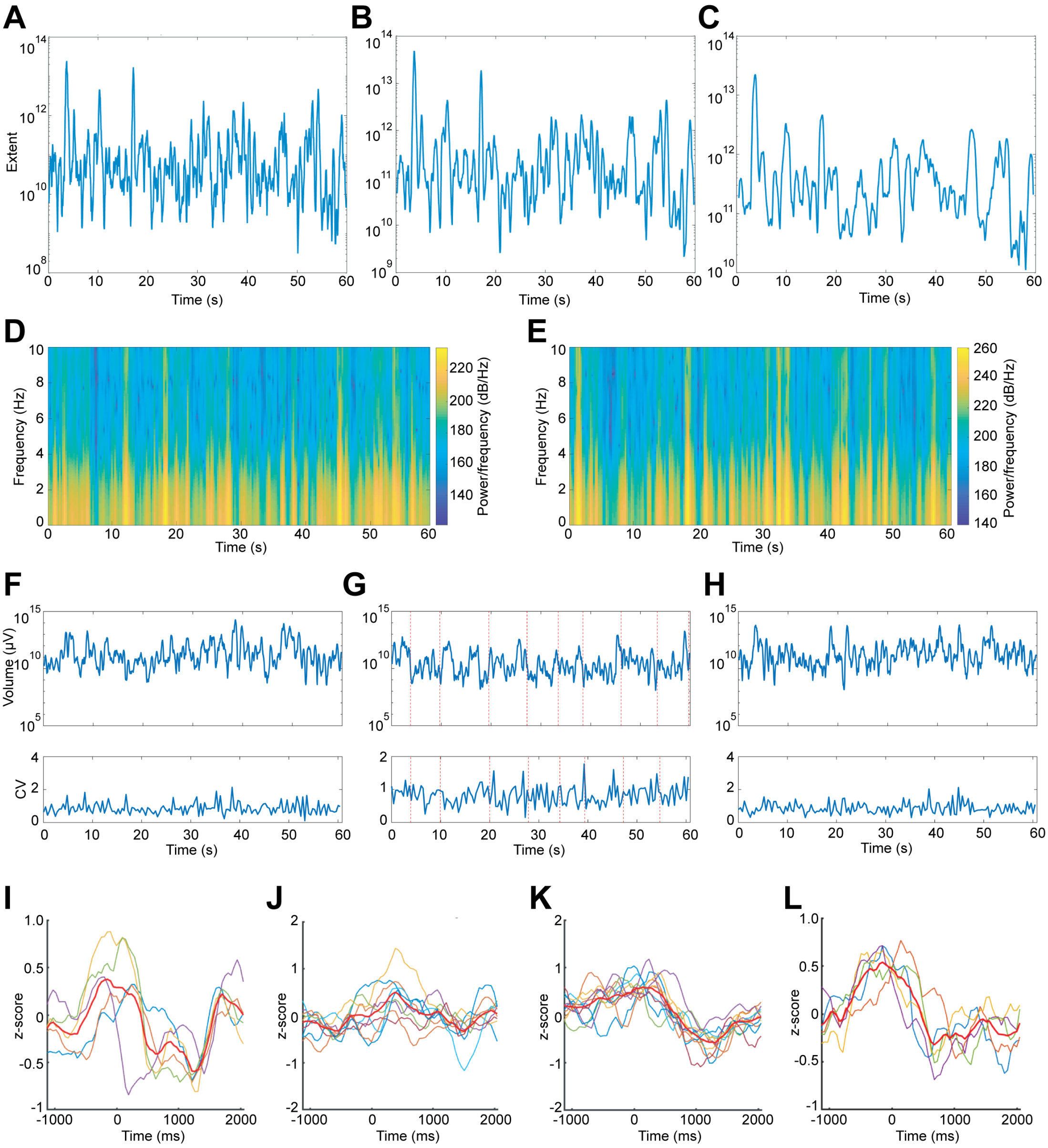

Fig. S5. Expansions and contractions of the volume of evolving states. Panels **A** to **H** show the volume (V) calculated according to equation 4 (Methods) to show fast fluctuations of the volumes**.** V is thus calculated as the volume of 25 consecutive cortical states. **A.** The evolution of V with a sliding window width of 128 states. **B.** Sliding window width of 256 states. **C.** Sliding window width of 512 states. **D.** Power spectrum of fluctuations of expansions and contractions of the volumes, classification of nouns (patient 12, lead 24), supramarginal gyrus. **E.** Power spectrum, judging the mood of faces (patient 17, lead 2), amygdala. **F.** V, and for comparison, the coefficient of variance of the z-score of $\log_{10}V$, CV (patient 8) rest, anterior hippocampus. **G.** Same patient, same electrode, classification of nouns, red vertical lines mark the peaks of the sounds of the nouns. **H.** Same patient, mental navigation (test 4), fusiform gyrus. F, G, H show typical ranges of the CV of $\log_{10}V$ between 0.5 and 2.5. However, the irregularity of V itself is orders of magnitude larger. **I.** Cross-correlated leads, z-scores (patient 14) recalling places. Different leads have different colors. The mean of the average z-scores is shown in red. **J.** Non-significant leads (patient 14) recalling places. **K.** Cross-correlated leads (patient 5) judging the moods of faces. **L.** Cross-correlated leads (patient 12) classifying nouns.

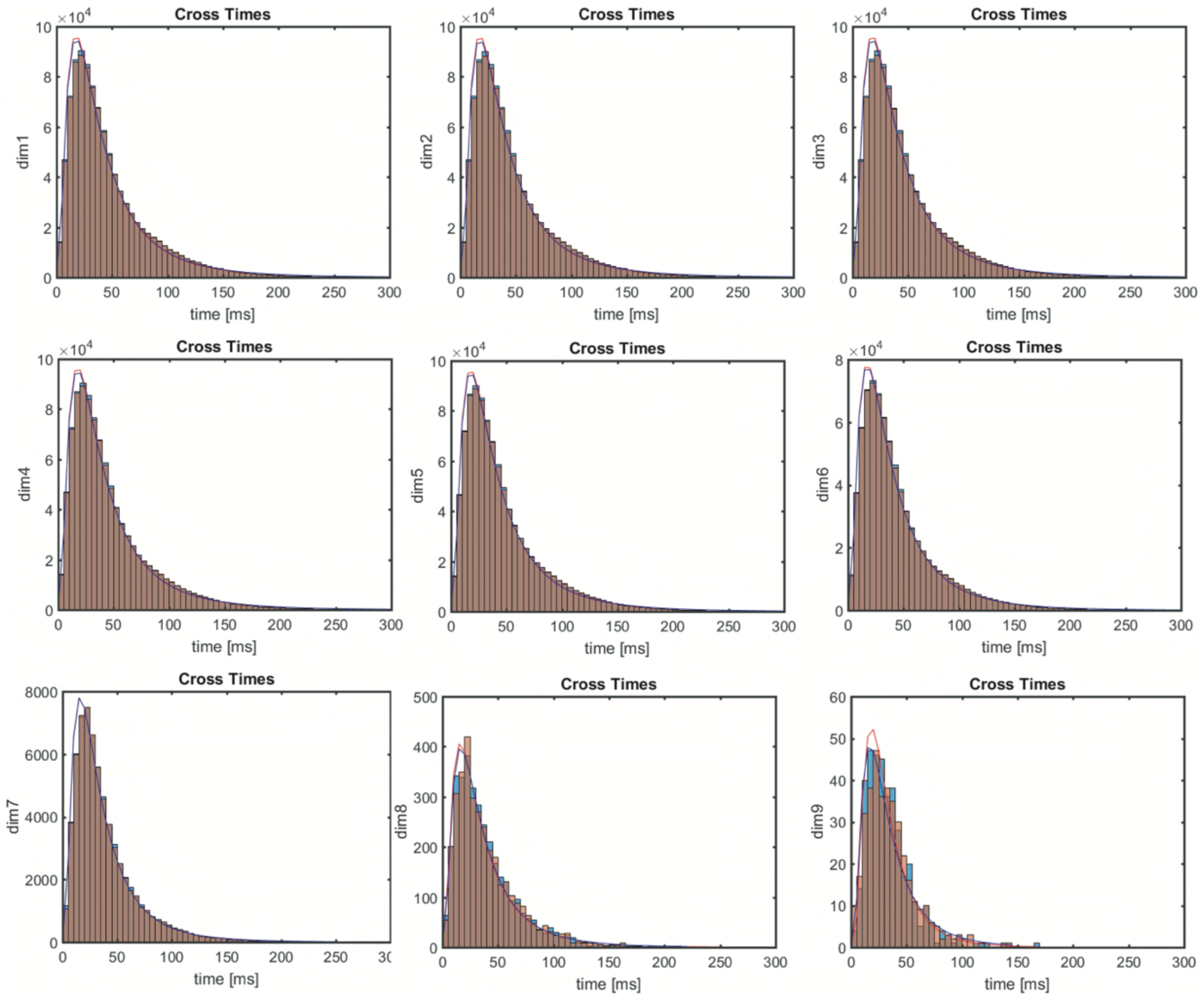

Fig. S6. Flow of all states with respect to symmetry planes. The flows of cortical states in the region of state space they occupy reveal the details of the dynamic. The panels show the distributions of the time the states spend in one sector until they cross the symmetry plane and continue into a new sector of the region, for all 3476 data sets. **Figure S4 C** and **E** show examples of regions with symmetry planes. Light brown: flow from the positive side to the negative; blue: states flowing from the negative to the positive side; brown overlap of the two distributions. All distributions were logarithmic normal (continuous curve: best fit). Median values for dimension 1 to 6 were 34 or 35 ms, for dimensions 7,8, and 9 30, 31 and 34 ms respectively. This together with Fig.3 limits the possibilities of alternative interpretations of the data (for example fixed point attractors, bifurcations, limit cycles, tori, multi-stabilities and longer lasting trajectory condensations).

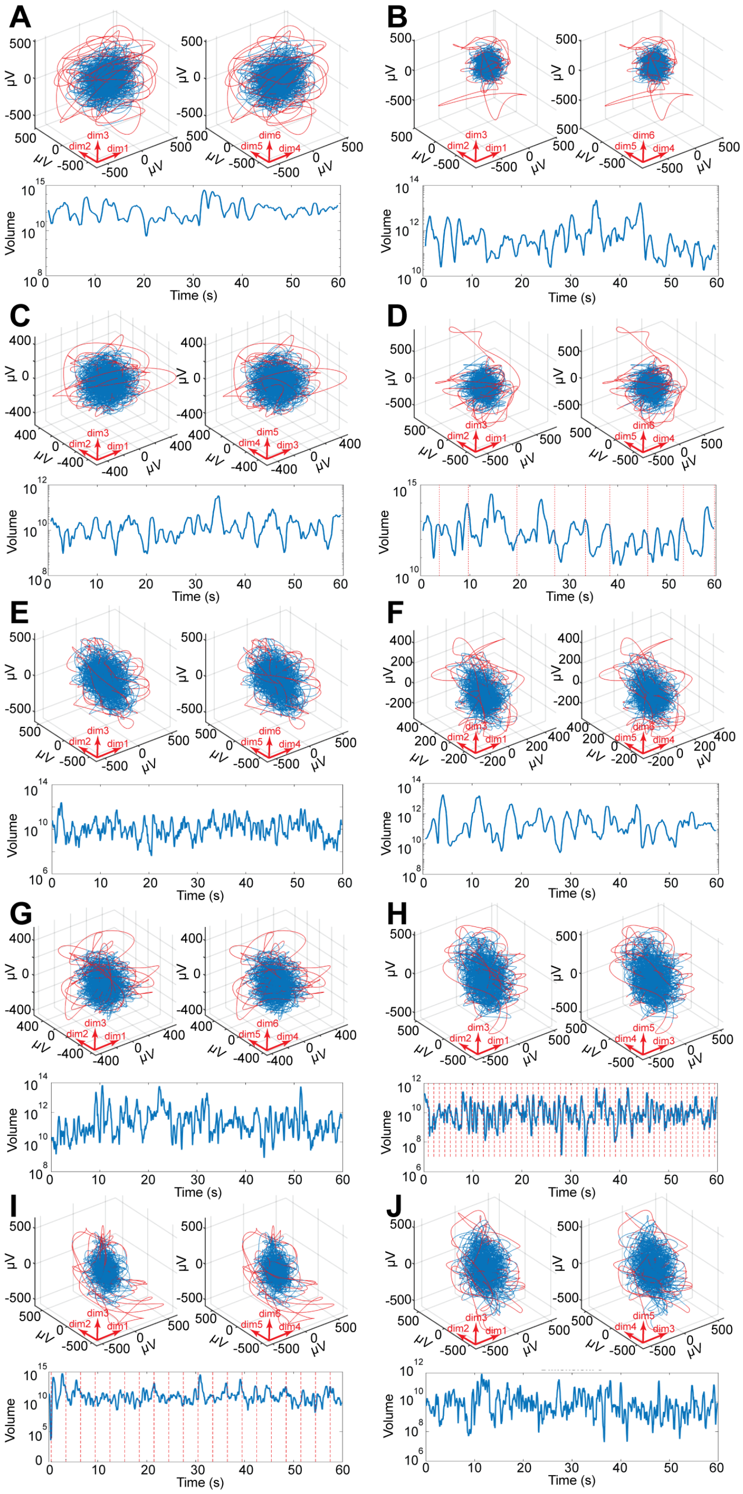

Fig. S7. Different brain functions emerge under the same type of dynamic. Projections of trajectories over 60s; Red curves: trajectories temporarily exploiting nearby state space. Blue curves: trajectories inside the state space defined by the enclosing hyper-elliptic volume $V_{95\%}$ (**Methods**). Below: fluctuations of the volume of cortical states (equation 4, **Methods**) during the same 60 s. **A.** Control (rest) condition, superior temporal gyrus, anterior part (patient 4). **B.** Mental navigation (memory retrieval test 8), posterior hippocampus (patient 6). **C.** Memory retrieval test 10 (admission day), frontal operculum (patient 12). **D.** Classification of nouns, parahippocampal gyrus (patient 8). **E.** Somatosensory attention, anterior insula (patient 16). **F.**

Watching TV, superior frontal gyrus, anterior part (patient 9). **G.** Visual motion to right (visual test 7), posterior hippocampus (patient 15). **H.** Visual test 4, middle part of inferior frontal gyrus (patient 14). Note entrainment. **I.** Recognition (visual test 6), subiculum (patient 10). **J.** Taste, amygdala (patient 16).

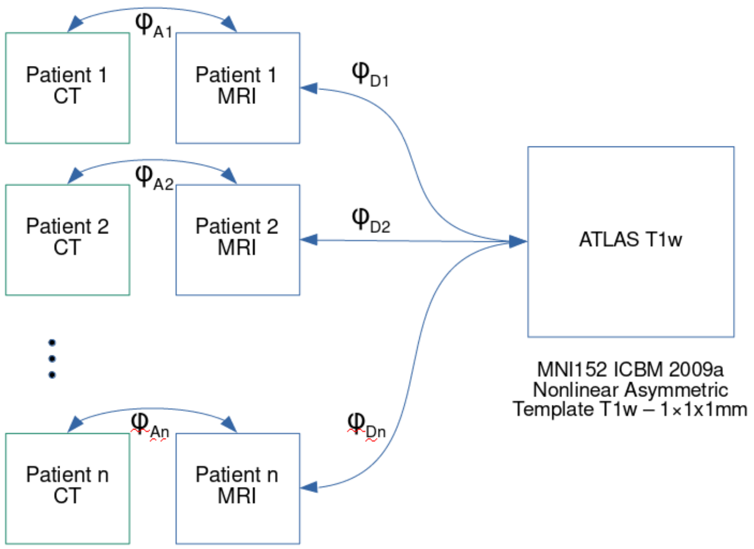

Fig. S8. Image registration pipeline using MNI152 ICBM 2009. The template used was the nonlinear asymmetric T1-weighted ICBM 2009, with a resolution of 1x1x1mm (1). The CT and MR images were registered with an affine transformation ϕAi, where i = 1, ..., n refers to the patients. The affine registration algorithm is intensity-based and implemented with ITK (2). The similarity metric is mutual information (3). The algorithm computes a rigid transformation first, and then the affine transformation in a multi-resolution framework. The affine transformation was applied to the CT images to align them to the MR images. Prior to atlas registration, the MRI images were segmented. Brain extraction was performed using the ROBEX algorithm (4). The MRI images were registered to the atlas with a deformable transformation, ϕDi. The deformable image registration method used was Symmetric Image Normalization (SyN) (5) which uses cross-correlation (CC) as similarity metric and the L2 norm of the velocity field as regularization. The transformation or displacement field ϕDi was computed by integration. Finally, the deformable transformation was applied to the CT and MR of each image patient.

Supplementary References:

1. Fonov, V.S., Evans, A.C., McKinstry, R.C., Almli, C. and Collins, D. (2009). Unbiased nonlinear average age-appropriate brain templates from birth to adulthood. NeuroImage 47, S102.

2. ITK. Insight segmentation and registration toolkit. Web Page: https://itk.org/, 2019. [Consulted in June 2019].

3. Thévenaz, P. and Unser, M (2000). Optimization of mutual information for multiresolution image registration. IEEE transactions on image processing, 9, 2083-2099.

4. Iglesias, J.E., Liu, C.-Y., Thompson, P.M. and Tu, Z. (2011). Robust brain extraction across datasets and comparison with publicly available methods. IEEE transactions on medical imaging, 30, 1617-1634.

5. Avants, B.B., Epstein, C.L., Grossman, M. and Gee, J.C. (2008). Symmetric diffeomorphic image registration with cross-correlation: evaluating automated labeling of elderly and neurodegenerative brain. Medical image analysis, 12, 26-41.

Table S1. Single trial tasks

Patient	Test	Active electrode leads
4	visual test 4	1,4,10,15,16,18, 21,22,23,29,33,37,40,49,53,54,57,65,68
	visual test 5	4,18,21,22,29,37,409,57,68
	visual test 6	1,4,10,15,16,18,21,22,28,29,33,49,57.65,68
	visual test 11	4,10,15,18,20,28,37,40,49,57,65,68
	emotions	49,53,54
	recall faces	1,22,37,40,57,68
	recall places	21,23,40,49,57
5	visual test 4	1,2,5,6,7,10,11,14,18,19,24,25,26,39,40,49,54,55,56,57,59,65
	visual test 5	1,10,11,14,18,19,24,25,26,54,55,56,57,65
	visual test 6	7,10,11,14,18,19,24,25,26,39,40,49,55,56,57,59,65
	visual test 11	1,25,26,39,40,54,65
	emotions	1,2,5,6,10,11,24,25,55,56,65
	recall faces	1,5,6,7,11,14,18,24,26,39,55,56,65
	recall places	1,18,26,54,65
6	visual test 4	1,2,3,15,17,51,57,105,115,116,117
	visual test 5	1,2,3,8,15,17,26,51,57,105,115,117
	visual test 6	1,2,3,15,51,57,58,105,107
	emotions	67,105,107,115
	recall faces	1,2,3,8,15,2651,57,105,115
8	visual test 5	9,26
	visual test 6	69,70,79
	recall faces	2,20
	recall places	73,96,97,98,99
	nouns	66,69,70,74,79
9	recall faces	21,61
	nouns	21,22,61
10	recall places	39,42
	nouns	12,39
11	visual test 4	36,41,46,47,48,50,53,56,68,69,88,101
	visual test 5	1,2,9,21,23,24,33,34,46,47,48,49,50,53,56,68,69,88
	visual test 6	8,10,21,23,24,25,41,46,47,48,49,50,53,68,69,88,93,101,102
	visual test 11	2,8,9,10,24,33,34,35,46,47,48,49,56,68,69
	emotions	24,25,35,43
	recall faces	23,24,88,101
	recall places	24,46,49,56
	nouns	20,50,53,88
12	visual test 4	1,2,6,10,14,15,19,22,23,24,25,26, 28,33,39,40,41,42,43,44,47,48,51,52,57,60,63,75,79,87,91,92,95,99
	visual test 5	2,6,10,14,15,19,22,23,24,25,26 28,33,39,40,41,42,43,44,47,48,51,52,57,60,63,75,79,87,92,95
	visual test 6	1,10,41,42,44,47,87,91
	visual test 11	1,26,41,87,91
	emotions	2,10,24,87,91,92
	recall faces	2,10,22,23,25,26,63,91
	recall places	10,22,33,41,42,43,45,46,91
	nouns	10,25,91,92,95
13	visual test 4	14,15,40,51
	visual test 5	17,40
	emotions	40
	recall faces	40
	recall places	40
14	visual test 4	26,40,62
	visual test 5	48,61,62
	visual test 6	8,40,48,50,61,62,63,69,79,80
	recall faces	61,62,63
	nouns	61,62,63
15	visual test 4	1,2,3,14,15,19,23,48,49,50
	visual test 5	1,3,11,14,15,23,46,50
	recall faces	3,11,14,15,19,23
	recall places	11,19,23,41
	nouns	11,14,15
16	emotions	1,11,19
	nouns	1
17	visual test 5	6,8,9,10,13,15,27,28,32,35,39,40,41,42,57,58,59,70,71,72,79,83,87,89
	visual test 11	9,13,15,28,32,33,35,39,57,58,59,70,71,72,83,87
	emotions	6,8,13,24,28,31,32,35,41,42,50,58,71,72,83,87,89
	recall faces	2,3,6,8,18,24,28,31,32,33,35,39,50,71,72,83,87
	recall places	59,70,72,83,89
	nouns	50,51,52,53,89

For each patient, electrode leads have numbers uniquely defining the cortical locations.
